# The prevalence of psychiatric comorbidities in adult ADHD compared with non-ADHD populations: A systematic literature review

**DOI:** 10.1371/journal.pone.0277175

**Published:** 2022-11-04

**Authors:** Won-Seok Choi, Young Sup Woo, Sheng-Min Wang, Hyun Kook Lim, Won-Myong Bahk

**Affiliations:** 1 Department of Psychiatry, Hallym University Sacred Heart Hospital, Anyang, Korea; 2 Department of Psychiatry, College of Medicine, The Catholic University of Korea, Seoul, Korea; Shinshu University School of Medicine, JAPAN

## Abstract

Comorbid psychiatric disorders in adults with ADHD are important because these comorbidities might complicate the diagnosis of ADHD and also worsen the prognosis. However, the prevalence of comorbid psychiatric disorders in adult ADHD varies according to the diagnostic tools used and the characteristics of target populations. The purpose of this review was to describe the prevalence of comorbid psychiatric disorders in adults with ADHD compared with adults without ADHD. Thirty-two studies published before August 2022 were identified and classified according to diagnosis of other psychiatric disorder in those with ADHD. The most frequent comorbid psychiatric disorder in the ADHD group was substance use disorder (SUD), followed by mood disorders, anxiety disorders, and personality disorders. The prevalence of these four disorders was higher in the ADHD group, whether or not subjects were diagnosed with other psychiatric disorders. In addition, the diversity of ADHD diagnostic tools was observed. This also might have affected the variability in prevalence of comorbidities. Standardization of ADHD diagnostic tools is necessary in the future.

## Introduction

ADHD(attention-deficit/hyperactivity disorder) is a common psychiatric disorder presenting persistent inattention and/or with hyperactivity/impulsivity [1], which is associated with considerable problems in personal, social, and occupational areas [2]. While ADHD is well studied in children, it is recently being studied in adults as well. According to a previous meta-analysis, 65% of children who were diagnosed with ADHD have persistent ADHD symptoms in adulthood [3]. In addition, the prevalence of ADHD in adults is known to reach 2.5% [4], which is moderate compared to its prevalence in children, which is about 5% [5].

Although comorbid psychiatric disorders are common in both adults and children, the comorbidity rate is higher in adults; as many as 80% of adults with ADHD are reported to have at least one comorbid psychiatric disorder [6–8]. In clinical adult ADHD samples, substance use disorder (SUD), mood disorder, anxiety disorder, and antisocial personality disorder (ASPD) are the most common comorbid disorders [9, 10], and these mental disorders can adversely affect patient prognosis. Furthermore, research revealed that comorbid psychiatric disorders cause considerable functional impairment in individuals with ADHD and place a great burden on society [11].

For this reason, several cross-sectional studies have been conducted on various populations including clinical and general settings over 30 years to evaluate the prevalence of comorbid psychiatric conditions in adults with ADHD. However, the prevalence of comorbid psychiatric disorders varied according to characteristics of the subjects, including country, race, gender, and other socioeconomic characteristics as well as the screening or diagnostic tools applied [10, 12]. Moreover, since ADHD has been recognized in adults, diagnostic tools for adult ADHD and its comorbid disorders have changed over time [13], and the interest in clinical diagnoses and optimal treatments in adults with ADHD has also increased [14]. These factors might have contributed to the divergent prevalence rate of ADHD and comorbid disorders in adults.

However, to the best of our knowledge, despite the high prevalence of psychiatric disorders documented in previous studies in the adult ADHD subjects [10, 11, 15, 16] and their importance in the clinical field, no systematic literature review has specifically compared the prevalence of comorbid psychiatric disorders between adults with and without ADHD. Considering the high prevalence of adult ADHD and its impact on quality of life, a perspective on frequent comorbid psychiatric disorders would be helpful for individuals with ADHD and clinicians. Thus, the aim of our study was to ascertain the difference in the prevalence rates of comorbid psychiatric disorders between adults with and without ADHD including both clinical and general populations.

## Methods

### Study search and data sources

The methodology of the present review followed the Preferred Reporting Items for Systematic Reviews and Meta-Analysis (PRISMA) [17]. Following PRISMA guideline, we conducted research based on PICO (Population, Intervention, Comparison and Outcome). The target population was adults with diagnosed ADHD. We compared the prevalence of comorbid psychiatric disorders between ADHD and non-ADHD patients. We searched electronic libraries of PubMed, EMBASE, PsycINFO, PsycNET, and Google Scholar for publications regarding the epidemiology and prevalence rate of comorbidities of adult ADHD published from 1 January 1990 to 1 August 2022. The initial search was conducted by two authors (WSC, YSW) using the following terms: Prevalence AND (ADHD OR ADD OR Attention Deficit) AND Adult AND (comorbidity OR comorbid) in titles or abstracts. Each database was updated as appropriate when preparing this submission for publication. Full electronic search strategies are provided in the S1 Text.

### Study selection

First, articles obtained from the initial search were de-duplicated by EndNote 20. Then, inclusion/exclusion screening was performed by lead authors (WSC, YSW) based on exclusion criteria of non-relevant articles (e.g., did not focus on adult patients or did not include psychiatric comorbidity data), non-English articles, full text not available, abstract-only papers, and articles that were not peer-reviewed. We included all types of research except a systematic review or meta-analysis defined by title and method. The initial inclusion/exclusion review was based on titles and abstracts; if the relevance of the article was unclear, a full-text review was performed to determine the eligibility of each study. After this initial process, the full texts of all included articles were retrieved to evaluate our detailed eligibility criteria. Articles were included in the study if they 1) used samples of adult populations aged 18 years or older, 2) defined clear ADHD and non-ADHD groups by clinically diagnoses or using any diagnostic criteria (e.g., DSM (Diagnostic and Statistical Manual of Mental Disorders)) or tools for screening/diagnosing ADHD in adults (e.g., ASRS (Adult ADHD Self-report Scale)), 3) defined the prevalence rate of comorbid psychiatric disorders using any diagnostic tools for each psychiatric disorder (e.g. SCID (Structured Clinical Interview for DSM-IV)), and 4) directly compared ADHD and non- ADHD groups using statistical analysis. Any discrepancy between the two lead authors during study selection was resolved through discussion, and other authors were consulted if necessary. The inclusion consistency between the two authors was 94.1% (32/34).

### Data collection process

Microsoft Excel was used to develop a data extraction spreadsheet, and all included full-text articles were reviewed by both researchers (WSC, YSW), who also conducted the initial data search and study selection process. The extracted data were reviewed for consistency, and any queries that arose were resolved by discussion among the researchers. The lead author decided whether to include/exclude data by reviewing the specific articles.

### Measurements

Because there are various methods for diagnosing ADHD and psychiatric disorders in adults, we extracted the following variables from the articles ultimately included: 1) data describing the study characteristics, such as year of publication, country, or study design; 2) data describing the target population, like sample size, age range or mean age/SD, or gender composition; 3) diagnostic tools for adult ADHD and comorbid psychiatric disorders, whether clinical diagnosis was performed, and the diagnostic criteria for ADHD/psychiatric comorbidity; 4) study results including the prevalence rate of ADHD in the target population, prevalence rate of each psychiatric comorbidity in each ADHD and non-ADHD group, and any statistically significant comparable variables including odd ratios(ORs) with 95% confidence intervals or chi-square (χ^2^) test variables.

### Classification of studies

Based on several studies targeting nation-wide psychiatric comorbidities [18–20], assuming that the prevalence of comorbid psychiatric disorders is higher in groups of psychiatric patients, we decided to divide the study populations into general population group studies and clinical group studies. A clinical group study was defined as one in which the study population included patients who had previously been clinically diagnosed with any psychiatric disorder or had visited/been admitted either voluntarily or involuntarily to a hospital for treatment. A general group study was defined as that in which the whole study population was not diagnosed with any psychiatric disorders before the start of each study.

In addition, considering the specificity that the prevalence of ADHD among incarcerated people was five to 10 times higher than that of the general population [21], and that the prevalence of comorbid psychiatric disorders among inmates was higher than that of the general population [22], we classified data of incarcerated patients separately from other population groups. The incarcerated group study was defined as a study that only included incarcerated participants.

## Results

**Fig 1:**
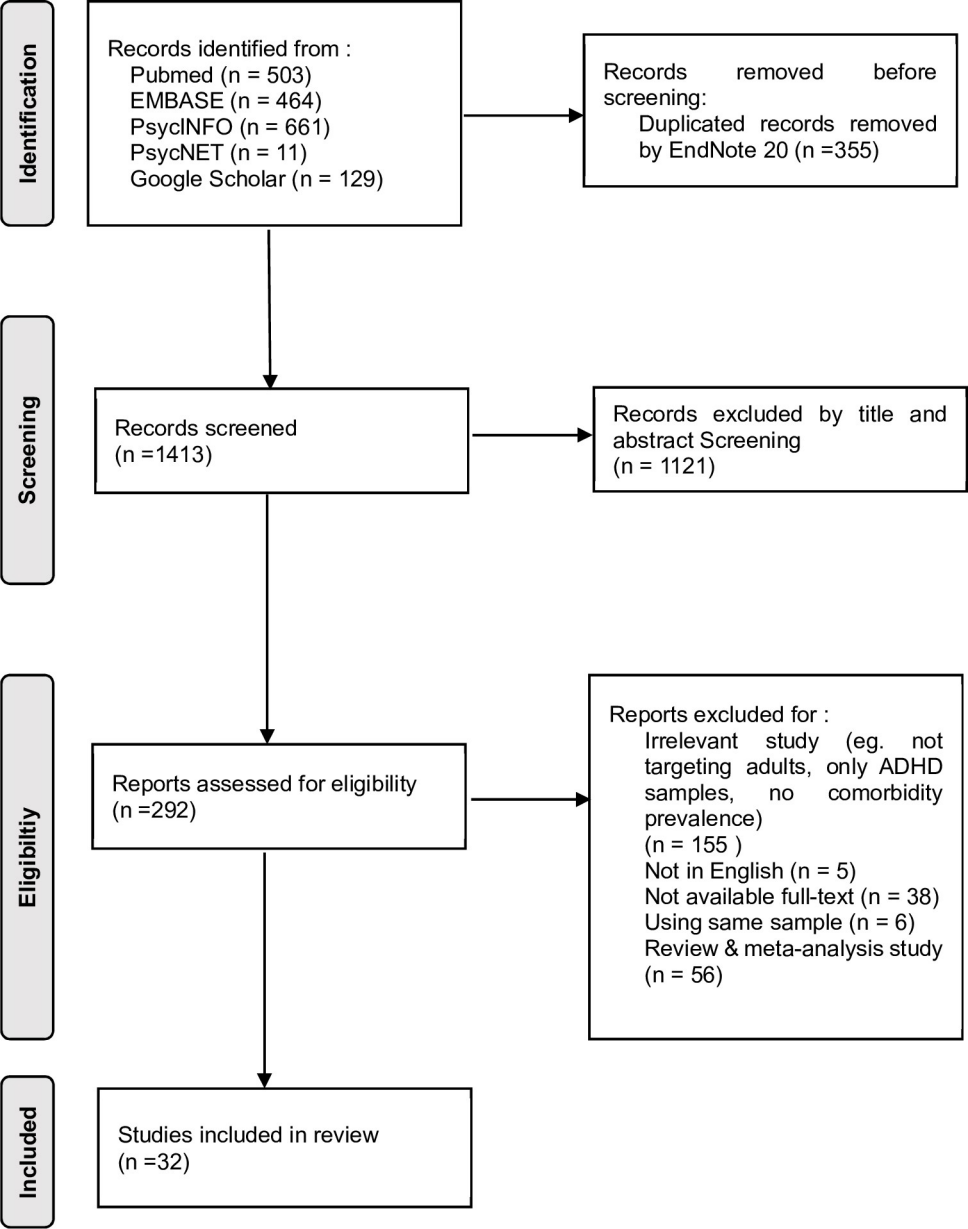
PRISMA flow diagram. Flow diagram of the manual screening process for eligible literature.

In total, 1768 articles were identified by the search method described above, and 335 duplicates were removed. After the duplicates were excluded, an additional 1121 articles were excluded by screening titles and abstracts. The remaining 292 articles were read in full and included in the analysis if they met the inclusion criteria of our study. Based on our study criteria, 260 articles were excluded for reasons noted in Fig 1. Thus, 32 studies comparing the prevalence rates of comorbid psychiatric disorders between ADHD and non-ADHD adult subjects were selected for systematic review.

Of the 32 studies comparing the prevalence of comorbid psychiatric disorder between subjects with and without adult ADHD, according to our classification criteria, 11 studies involved general populations, 18 studies included psychiatric populations, and three studies focused on incarcerated populations. One of the three studies dealing with incarcerated populations involved only female inmates [23], and the other one study involved only male inmates [24].

### Diagnostic tools of included studies

In this review, 12 diagnostic tools including clinical diagnostic criteria like DSM or ICD(International Classification of Disease), were used to evaluate adult ADHD. In addition, five diagnostic tools were mainly used for comorbid psychiatric disorders. The most used evaluation tool for adult ADHD was Adult ADHD Self-Report Scale (ASRS) [25, 26], which was used in 12 studies. Five studies used the ASRS alone to evaluate ADHD in adults, and the rest of the studies used more than one tool to evaluate ADHD. The next most frequently used evaluation tools were Conner’s Adult ADHD Diagnostic Interview for DSM-IV(CAADID) [27] and the Wender Utah Rating Scale (WURS) [28].

### Prevalence of mood disorders

Nineteen studies provided data comparing the prevalence of mood disorders (including depressive disorders and bipolar disorders) between ADHD patients and non-ADHD individuals [6, 16, 29–45]. In the general populations, the prevalence of any depressive disorder in the non-ADHD group was estimated at 1.2% [16] to 12.5% [35], compared to 8.6% [36] to 55% [6] in the ADHD group. In clinical populations, the prevalence of any depressive disorder in the non-ADHD group was estimated at 5.8% [32] to 39.6% [34], compared to 15.4% [32] to 39.7% [44] in the ADHD group. In the general population, the prevalence of any bipolar disorder in the non-ADHD group was estimated at 0.2% [35] to 3.6% [16] compared to 4.48% [42] to 35.3% [16] in the ADHD group. In clinical populations, the prevalence of any bipolar disorder in the non-ADHD group was estimated at 2.0% [37] to 19.5% [40], compared to 7.4% [34] to 80.0.% [29] in the ADHD group. There were no differences reported in the prevalence of mood disorders between ADHD and non-ADHD groups in the incarcerated population studies. Detailed information from each study is summarized in Table 1.

**Table 1:** Studies comparing the prevalence of mood disorders between non-ADHD and ADHD subjects.

Author	Year,	Country	N	% of male	Age	Assessment of ADHD	Assessment of comorbid psychiatric disorder	Design	Sample	Prev.of ADHD(%) (non-ADHD/ADHD)	Findings comparing non-ADHD and ADHD and prevalence of comorbid psychiatric disorders	non-ADHD, n (%) vs ADHD, n (%)
General sample												
Solberg et al [43]	2018	Norway	1,701,206	51.2%	18≤	ADHD medication at adult or ADHD diagnosis registered	ICD-10	Cross-sectional study	General sample	2.4% (40,103/1,661,103)	Bipolar Disorder	Women 13,183 (1.6%) vs 2,290 (12.9%) Men 9,009 (1.1%) vs 1,981 (8.9%)
											Major depressive disorder	Women 61,880 (7.6%) vs 5,138 (28.8%) Men 33,733 (4.0%) vs 4,516 (20.3%)
Chen et al [31]	2018	Norway	5,551,807	50.81%	18–64	ICD-9: 314; ICD-10: F90 diagnosis	ICD	Cross-sectional study	General sample	1.1% (61,129/5,490,678)	Depression	PR = 9.01 (8.92–9.10)
											Bipolar Disorder	PR = 19.96 (19.48–20.43)
Hesson and Fowler [35]	2018	Canada	16,957	NA	20–64	Self-report of ADHD (diagnosed by a health professional)	WHO-CIDI modified for the needs of CCHS-MH	Case-control study	General sample -national mental health survey	2.9% (NA)	12-month Major depressive disorder	61 (12.5%) vs 113 (23.3%) χ2 = 59.94
											12-month Bipolar disorder	1 (0.2%) vs 20 (4.1%) χ2 = 17.73
Yoshimasu et al [16]	2016	US	5,718	NA	Mean age ADHD 30.2 (SD 1.9) Non-ADHD controls 30.2 (SD 2.0)	Childhood-identified ADHD with M.I.N.I (+)	M.I.N.I	Case-control study	General population–Birth cohort sample	NA, (68/335)	Hypomanic episode—current or past	12 (3.6%) vs 24 (35.3%) OR adj 16.5 [7.2, 37.4]
											Dysthymia	4 (1.2%) vs 11 (16.2%) OR adj 19.0 [5.4, 66.1]
											MDD	9 (2.7%) vs 19 (27.9%) OR adj 15.2 [6.2, 37.4]
Park et al [39]	2011	South Korea	6,081	ADHD+ 59.4% ADHD- 50.5%	18–59	ASRS-S v 1.1 (+)	K-CIDI (Korean Ver. of CIDI)	Epidemiological study	General sample	1.1% (69/6,012)	Any mood disorder	6.0% vs 27.1% OR 6.44 [3.70–11.19]
											Major depressive disorder	5.5% vs 17.4% OR = 4.00[2.10–7.63]
											Bipolar disorder	0.2% vs 8.6% OR 29.94 [10.71–83.66]
Miller et al [38]	2007	US	363	51.0%	18–37	K–SADS & structured interview	SCID–I, SCID-II	Case-control study	General sample - Recruited ADHD vs control group	NA, (152/211)	Mood disorder	NA, χ2 = 23.70
Sobanski et al [6]	2007	Germany	140	54.3%	Mean age ADHD+ 36.8 (SD 9.0) ADHD- 39.8 (SD 10.0)	WURS-K & BADDS	SCID-I	Case-control study	General sample -referred ADHD vs control group	NA, (70/70)	Affective disorders total	18 (25.7%) vs 44 (60.7%) χ2 = 18.462
											Major depressive episodes	17 (24.3%) vs 40 (55%) χ2 = 15.010
Kessler et al [36]	2006	US	3,199	NA	18–44	DIS-IV for childhood pathology & ACDS v 1.2 (ADHD-RS)	CIDI	Epidemiological study	General sample–national survey	2.6% (NA)	Major depressive disorder	7.8%vs 8.6% 4.2 OR 2.7[1.5–4.9]
											Dysthymia	1.9% vs 12.8% OR 7.5 [3.8–15]
											Bipolar	3.1% vs 19.4% OR 7.5 [4.6–12.0]
											Any mood disorder	11.1% vs 38.3% OR 5.0 [3.0–8.2]
Secnik et al [42]	2005	US	4,504	64.3%	18≤	ICD-9	ICD-9	Case-control study	General sample–HPM database	(2,252/2,252)	Bipolar disorder	0.58% vs 4.48%
											Depression	2.93% vs 17.10%
Clinical sample												
Woon and Zakaria [45]	2019	Malaysia	120	94.2%	18–65	CAADID	M.I.N.I	Cross-sectional study	Psychiatric sample	15.8% (101/19)	Manic/hypomanic episode, lifetime	8 (7.9%) vs 8 (42.1%)
Roncero et al [41]	2019	Spain	726	72.5%	18≤	ASRS (14≤)	DSM-IV-TR	Cross-sectional study	Psychiatric sample–treatment seeking AUD patients	21.1% (573/153)	Mood disorder	24.5% vs 49% χ^2^ = 32.87, OR 2.95 [2.2, 4.3]
Leung and Chan [37]	2017	Hong Kong	254	28.7%	18–64	ASRS-v1.1 Symptom Checklist≥17 & SDS ≥5 (Screening) + DIVA 2.0 (Diagnosis)	DSM-5	Cross-sectional cohort study	Psychiatric sample–clinical outpatients	19.3% (49/205)	Bipolar disorder	2.0% vs 15.0% OR = 8.87 (1.83–42.9) (ADHD-combined type vs Non-ADHD)
Gorlin et al [34]	2016	US	1,134	42%	Mean age 39.7 (SD 14.4)	DSM-IV based semi-structured clinical interview	SCID	Cross-sectional study	Psychiatric sample—clinical outpatients	18.0% (204/903)	Major depressive disorder	39.6% vs 29.4% OR = 0. 69 (0.49–0.96)
											Bipolar disorder	3.4% vs 7.4% OR = 2.14 (1.09–4.02)
Fatséas et al [33]	2016	France	217	66.4%	18–65	CAADID	DSM-IV for SUD SCIDII for BPD M.I.N.I. for others	Cross-sectional cohort study	Psychiatric sample–addiction clinical outpatients	23.0% (50/167)	Current mood disorders	36.8% vs 54.0% 0.030
van Emmerik-van Oortmerssen et al [44]	2014	Australia, Belgium, France, Hungary, Netherlands, Norway, Spain, Sweden, Switzerland, US (IASP study) (37)	1,205	ADHD– 73.1%ADHD + 75.6%	18–65	CAADID	MINI Plus SCID-II	Cross-sectional study	Psychiatric sample—treatment-seeking SUD patients	13.9% (168/1,037)	Current Depression—alcohol	15.3% vs 39.7% OR 4.1 [2.1–7.8]
											Current (hypo)mania	4.1% vs 14.9% OR 4.3 [2.1–8.7]
Duran et al [32]	2014	Turkey	246	NA	18–60	WURS score >36 & Turgay’s Adult ADD/ADHD Evaluation Scale	SCID-I-CV, SCID-II	Cross-sectional study	Psychiatric sample—clinical outpatients	15.9% (39/207)	Dysthymic Disorder	12 (5.8%) vs 6 (15.4%) χ2 = 25.81
Perugi et al [40]	2013	Italy	96	59.4%	18–65	ASRS v 1.1 (+), & prior age 7 with ADHD sx	DSM-IV	Cross-sectional observation study	Psychiatric sample—Bipolar I, II disorder diagnosed	19.8% (19/77)	BD I mixed state	14 (18.2%) vs 10 (52.6%) χ2 = 9.6
											BD I mania	13 (16.9%) vs 0 (0%) χ2 = 3.7
											BD I remission	15 (19.5%) vs 0 (0%) χ2 = 0.1
Ceraudo et al [29]	2012	Italy	119	68.1%	Mean age ADHD+ 35.10 (SD 7.66) ADHD- 34.74 (SD 8.46)	ASRS-S v 1.1 (+)	DCTC (Diagnostic, Clinical and Therapeutic Checklist)	Cross-sectional study	Psychiatric sample – SUD outpatients	18.35% (20/89)	Bipolar Disorder	38 (43.2%) vs 16 (80.0%) χ2 = 8.84
											Mixed/Manic	15 (16.9%) vs 8 (40.0%) χ2 = 3.29
Olsson et al [30]	2022	Sweden	804	67.3%	18≤	ICD-10 or prescription of ADHD medication	ICD-10	Cross-sectional study	Psychiatric sample–psychiatric emergency patient with NSSI	11.6% (711/93)	Depression	218 (31%) vs 12 (13%), χ2 = 12.7

OR: Odd Ratio, PR: Prevalence Ratio, NA: Not available (not identified in article)

SUD: Substance Use Disorder, AUD: Alcohol use disorder, BPD: Borderline Personality Disorder BD I: Bipolar I disorder, WHO-CIDI: World Health Organization version of the Composite International Diagnostic Interview, CCHS-MH: Community Health Survey–Mental Health, M.I.N.I: Mini-International Neuropsychiatric Interview, ASRS-S: Adult Self-Report Scale-Screener, ASRS: Adult Self-Report Scale, CIDI: Composite International Diagnostic Interview, K-SADS: Kiddie Schedule for Affective Disorders and Schizophrenia, SCID: Structured Clinical Interview for DSM-IV, SCID-I: Structured Clinical Interview for DSM-IV Axis I Disorders, SCID-I-CV: Structured Clinical Interview for DSM-IV Axis I Disorders, Clinician Version, SCID-II: Structured Clinical Interview for DSM-IV Axis II Disorders, WURS: Wender Utah Rating Scale, WURS-k: German short form of the Wender Utah rating scale, BADDS: Brown attention deficit disorder scale, DIS-IV: Diagnostic Interview Schedule for DSM-IV, ACDS: Adult ADHD Clinical Diagnostic Scale, ASHD-RS: ADHD Rating Scale, CAADID: Conner’s Adult ADHD Diagnostic Interview for DSM-IV, ISAP: International ADHD in Substance use disorder Prevalence, NSSI: Nonsuicidal self-injury

### Prevalence of anxiety and related disorders

Sixteen studies provided data comparing the prevalence of anxiety disorders including obsessive-compulsive disorder, somatoform disorders and trauma/stress-related disorders between ADHD patients and non-ADHD individuals [12, 16, 31, 34–36, 38, 39, 41–43, 45–49]. In general population, the prevalence of any anxiety disorders in the non-ADHD group was estimated at 0.5% [39] to 9.5% [36] compared to 4.3% [39] to 47.1% [36] in ADHD group. In clinical populations, the prevalence of any anxiety disorders in the non-ADHD group was estimated at 5.4% [46] to 40% [49] compared to 3.9% [34] to 84% [49] in the ADHD group. Only one study of incarcerated populations showed a difference in the prevalence of social phobia between non-ADHD and ADHD individuals [47]. Detailed information from each study is summarized in Table 2.

**Table 2:** Studies comparing the prevalence of anxiety and related disorder between non-ADHD and ADHD subjects.

Author	Year	Country	N	% of male	Age	Assessment of ADHD	Assessment of comorbid psychiatric disorder	Design	Sample	Prev. of ADHD(%) (non-ADHD/ADHD)	Findings comparing non-ADHD and ADHD and prevalence of comorbid psychiatric disorders	non-ADHD, n (%) vs ADHD, n (%)
General sample												
Solberg et al [43]	2018	Norway	1,701,206	51.2%	18≤	ADHD medication at adult or ADHD diagnosis registered	ICD-10	Cross-sectional study	General sample	2.4% (40,103/1,661,103)	Anxiety Disorders	Women 54,479 (6.7%) vs 4,676 (26.3%) Men 28,364 (3.3%) vs 4,054 (18.2%)
Chen et al [31]	2018	Norway	5,551,807	50.81%	18–64	ICD-9: 314; ICD-10: F90 diagnosis	ICD	cross-sectional study	General sample	1.1% (61,129/5,490,678)	Anxiety	PR = 9.12 (9.04–9.21)
Hesson and Fowler [35]	2018	Canada	16,957	NA	20–64	Self-report of ADHD (diagnosed by a health professional)	WHO-CIDI modified for the needs of CCHS-MH	Case-control study	General sample -national mental health survey	2.9% (NA)	Generalized anxiety disorder	15 (3.1%) vs 73 (15.1%) χ2 = 42.30
Yoshimasu et al [16]	2016	US	5,718	NA	Mean age ADHD 30.2 (SD 1.9) Non-ADHD controls 30.2 (SD 2.0)	Childhood-identified ADHD with M.I.N.I (+)	M.I.N.I.	Case-control study	General population– Birth cohort sample	NA, (68/335)	PTSD	3 (0.9%) vs 6 (8.8%) OR adj. 10.0 [2.9, 35.0]
											Social phobia-current	4 (1.2%) vs 10 (14.7%) OR adj 12.8 [4.2, 39.4]
											OCD	8 (2.4%) vs 14 (20.6%) OR adj 8.0 [3.3, 19.2]
											Generalized anxiety disorder	30 (9.0%) vs 22 (32.4%) OR adj 4.7 [2.4, 9.0]
											Panic disorder–lifetime	17 (5.1) vs 9 (13.2) OR adj 2.6 [1.1, 6.2]
Park et al [39]	2011	South Korea	6,081	ADHD+ 59.4% ADHD- 50.5%	18–59	ASRS-S v 1.1 (+)	K-CIDI (Korean Ver. of CIDI)	Epidemiological study	General sample	1.1% (69/6,012)	Any anxiety disorder	6.3% vs 25.7% OR 5.46 [3.11–9.57]
											OCD	0.6% vs 4.3% OR 8.26 [2.51–27.26]
											PTSD	1.2% vs 7.2% OR 8.13[3.26–20.32]
											Social phobia	0.5% vs 11.4% OR 7.57 [1.92–29.83]
											Specific phobia	3.9% vs 11.4% OR 3.31 [1.52–7.18]
											Somatoform disorder	1.1% vs 4.3% OR 4.30 [1.22–15.12]
Miller et al [38]	2007	US	363	51.0%	18–37	K–SADS & structured interview	SCID–I, SCID-II	Case-control study	General sample- Recruited ADHD vs control group	NA, (152/211)	Anxiety disorder	χ2 = 8.81
Kessler et al [36]	2006	US	3,199	NA	18–44	DIS-IV for childhood pathology & ACDS v 1.2 (ADHD-RS)	CIDI	Epidemiologic study	General sample–national survey	2.6% (NA)	GAD	2.6% vs 8.0% OR 3.2 [1.5–6.9]
											PTSD	3.3% vs 11.9% OR 3.9 [2.1–7.3]
											Panic disorder	3.1% vs 8.9% OR 3.0 [1.6–75.9]
											Agoraphobia	0.7% vs 4.0% OR 5.5 [1.6–18.5]
											Specific phobia	9.5% vs 22.7% OR 2.8 [1.7–4.6]
											Social Phobia	7.8% vs 29.3% OR 4.9 [3.1–7.6]
											Any anxiety disorder	19.5% vs 47.1% OR 3.7 [2.4–5.5]
Secnik et al [42]	2005	US	4,504	64.3%	18≤	ICD-9	ICD-9	Case-control study	General sample–HPM database	NA (2,252/2,252)	Anxiety disorder	3.46% vs 13.77%
Clinical sample												
El Ayoubi et al [49]	2020	France	551	83.8%	18≤	Both ASRS-S v1.1(+) and WURS (26≤)	PCL-5 for PTSD	Cross-sectional study	Psychiatric inpatients with AUD	19.8% (442/109)	PTSD	179 (40%) vs 91 (84%) χ^2^ = 64.7
Woon and Zakaria [45]	2019	Malaysia	120	94.2%	18–65	CAADID	M.I.N.I	Cross-sectional study	Psychiatric sample -Forensic ward inpatient	15.8% (101/19)	Generalized anxiety disorder	20 (19.8%) vs 9 (47.4%)
Roncero et al [41]	2019	Spain	726	72.5%	18≤	ASRS (14≤)	DSM-IV-TR	Cross-sectional study	Psychiatric patients–treatment seeking AUD patients	21.1% (573/153)	Anxiety disorder	10.5% vs 25.8% χ^2^ = 23.5 OR 2.95 [1.88, 4.64]
Reyes et al [48]	2019	US	472	64.6%	18–80	PRISM	PRISM	Cross-sectional study	Psychiatric sample–inpatient & outpatient with DSM-IV-TR AUD diagnosis	6.36% (30/442)	Anxiety disorders, current	95 (21.5%) vs 14 (46.7%)
Gorlin et al [34]	2016	US	1,134	42%	Mean age 39.7 (SD 14.4)	DSM-IV based semi-structured clinical interview	SCID	Cross-sectional study	Psychiatric sample-clinical outpatient	18.0% (204/903)	Social phobia	28.7% vs 38.2% OR = 1.46 (1.05–2.01)
											Any adjustment disorder	9.4% vs 3.9% OR = 0.41 (0.18–0.82)
Retz et al [12]	2016	Germany	163	86.5%	Mean age 40.2 (SD 9.4)	DSM-5 & WURS-k ≥ 30	ICD-10	Cross-sectional study	Psychiatric sample–GD dx according to ICD-10	25.2% (41-current ADHD/122)	Stress and adjustment disorders	14 (8.6%) vs 7 (17.1%) χ2 = 5.70
Karaahmet et al [46]	2013	Turkey	90	53.3%	18≤	Turgay’s Adult ADD/ADHD Evaluation Inventory & WURS	SCID-I	Cross-sectional study	Psychiatric sample- Bipolar disorder diagnosed	23.3% (21/69)	OCD	6 (10.7%) vs 4 (19.0%)
											Panic disorder	3 (5.4%) vs 5 (23.8%)
Incarcerated sample												
Moore et al [47]	2016	Australia	88	76%	18–72	ASRS-S (+) & M.I.N.I plus (+)	M.I.N.I plus, PDQ-4, SCID-II	Cross-sectional study	Incarcerated sample	17.0% (15/73)	Social phobia	15.1% vs 46.7% OR = 4.39 [1.10, 17.56]

OR: Odd Ratio, NA: Not available (not identified in article)

PTSD: Post-traumatic stress disorder, OCD: Obsessive-compulsive disorder, GAD: Generalized anxiety disorder, AUD: Alcohol used disorder, GD: Gambling disorder WHO-CIDI: World Health Organization version of the Composite International Diagnostic Interview, CCHS-MH: Community Health Survey–Mental Health, M.I.N.I: Mini-International Neuropsychiatric Interview, ASRS-S: Adult Self-Report Scale-Screener, ASRS: Adult Self-Report Scale, CIDI: Composite International Diagnostic Interview, K-SADS: Kiddie Schedule for Affective Disorders and Schizophrenia, SCID-I: Structured Clinical Interview for DSM-IV Axis I Disorders, SCID-II: Structured Clinical Interview for DSM-IV Axis II Disorders, WURS: Wender Utah Rating Scale, WURS-k: German short form of the Wender Utah rating scale, PRISM: Psychiatric research interview for substance and mental disorders, PDQ-4: Personality disorder diagnostic questionnaire for the DSM-IV

### Prevalence of substance use disorders and gambling disorder

Twenty-two studies provided data comparing the prevalence of substance use disorders (including addiction to alcohol, opioids, stimulants, cannabis, anxiolytics, and nicotine) and gambling disorders between ADHD and non-ADHD individuals [6, 12, 16, 23, 24, 31–33, 35–42, 45, 47, 48, 50–52]. In general populations, the prevalence of any substance use disorder in the non-ADHD group was estimated at 0% [6] to 16.6% [39] compared to 2.3% [35] to 41.2% [16] in the ADHD group. In clinical populations, the prevalence of any substance use disorder in the non-ADHD group was estimated to be 2.0% [45] to 72.2% [41] compared to 10.0% [48] to 82.9% [41] in the ADHD group. Two studies compared the prevalence of gambling disorder between ADHD and non-ADHD patients, and there was one study for each general/psychiatric population group, showing a statistically significant difference in prevalence [37, 39]. Two studies of incarcerated populations showed differences in the prevalence of benzodiazepine use disorder [47] and drug dependence [23]. Detailed information from each study is summarized in Table 3.

**Table 3:** Studies comparing the prevalence of substance use disorder and gambling disorder between non-ADHD and ADHD subjects.

Author,	Year	Country	N	% of male	Age	Assessment of ADHD	Assessment of comorbid psychiatric disorder	Design	Sample	Prev.of ADHD(%) (non-ADHD/ADHD)	Findings comparing non-ADHD and ADHD and prevalence of comorbid psychiatric disorders	non-ADHD, n (%) vs ADHD, n (%)
General sample												
Cipollone et al [51]	2020	US	18,913	88.3%	Mean age 28.72 in non-ADHD, 28.56 in ADHD	ASRS-S (+)	CIDI & CIDI-SAM	Cross -sectional study (All army study)	General sample–Military sample	6.6% (17,674/1,239)	Previous 30-days SUD diagnosis	714 (4.04%) vs 211 (17.03%) χ^2^ = 515.36
											Lifetime SUD diagnosis	2,639 (14.93%) vs 503 (40.60%) χ^2^ = 780.16
											Alcohol use (type 2—Five or more drinks per day- heavy drinking)	2,064 (12.04%) vs 305 (25.10%) χ^2^ = 172.07
Capusan et al [50]	2019	Sweden	18,167	40.08%	20–45	DSM-IV criteria	SCID-I	Population-based epidemiological study	General population- Swedish Twin Registry	8.8% (1,598/16,569)	Alcohol abuse	OR = 1.88 [1.44, 2.46]
											Alcohol dependence	OR = 3.58 [2.86, 4.49]
											Stimulants	OR = 2.45 [1.79, 3.35]
											Opiates	OR = 1.97 [1.65, 2.36]
											Cannabis Illicit drug use	OR = 2.19 [1.80, 2.68] OR = 2.27 [1.86, 2.76]
											Poly-substance use	OR = 2.54[2.00, 3.23]
											Poly-substance use including alcohol	OR = 2.78 [2.21, 3.50]
Chen et al [31]	2018	Norway	5,551,807	50.81%	18–64	ICD-9: 314; ICD-10: F90 diagnosis	ICD	cross-sectional study	General sample	1.1% (61,129/5,490,678)	SUD	PR = 9.74 (9.62–9.86)
Hesson and Fowler [35]	2018	Canada	16,957	NA	20–64	Self-report of ADHD (diagnosed by a health professional)	WHO-CIDI modified for the needs of CCHS-MH	Case-control study	General sample -national mental health survey	2.9% (NA)	12-month Alcohol dependence	8 (1.7%) vs 27 (5.6%) χ2 = 10.83 .001
											Cannabis abuse	3 (0.6%) vs 13 (2.7%) χ2 = 6.376 .012
											Cannabis dependence	3 (0.6%) vs 11 (2.3%) χ2 = 4.605 .032
											Other drug dependence	3 (0.6%) vs 17 (3.5%) χ2 = 10.01 .002
Yoshimasu et al [16]	2016	US	5,718	NA	Mean age ADHD 30.2 (SD 1.9) Non-ADHD controls 30.2 (SD 2.0)	Childhood-identified ADHD with M.I.N.I (+)	M.I.N.I.	Case-control study	General population– Birth cohort sample	NA, (68/335)	Alcohol dependence/abuse	51 (15.2%) vs 28 (41.2%) OR adj 3.6 [2.0, 6.7]
											Substance dependence/abuse	22 (6.6%) vs 18 (26.5%) OR adj 4.4 [2.1, 9.1]
Park et al [39]	2011	South Korea	6,081	ADHD+ 59.4%ADHD- 50.5%	18–59	ASRS-S v 1.1 (+)	K-CIDI (Korean Ver. Of CIDI)	Epidemiological study	General sample	(69/6,012)	Alcohol abuse/dependence	16.6% vs 30.4% OR 1.97 [1.14–3.38]
											Nicotine dependence	7.7% vs 20.3% OR 2.81 [1.50–5.29]
											Pathological gambling	0.7% vs 1.4% OR 8.43 [2.63–26.96]
Miller et al [38]	2007	US	363	51.0%	18–37	K–SADS & structured interview	SCID–I, SCID-II	Case-control study	General sample- Recruited ADHD vs control group	NA, (152/211)	Any ADHD	SUD χ2 = 9.22
Sobanski et al [6]	2007	Germany	140	54.3%	Mean age ADHD+ 36.8 (SD 9.0) ADHD- 39.8 (SD 10.0)	WURS-K & BADDS	SCID-I	Case-control study	General sample -referred ADHD vs control group	NA, (70/70)	Substance related disorders total	5 (7.1%) vs 21 (30.0%) χ2 = 12.397
											Substances total	2 (2.9%) vs 20 (28.5%) χ2 = 17.806
											Substance abuse	2 (2.9%) vs 12 (17.1%) χ2 = 8.104
											Substance dependence	0 (0%) vs 8 (11.4%) χ2 = 8.612
Kessler et al [36]	2006	US	3,199	NA	18–44	DIS-IV for childhood pathology & ACDS v 1.2 (ADHD-RS)	CIDI	Epidemiologic study	General sample–national survey	2.6% (NA)	Drug dependence	0.1% vs 4.4% OR 7.9 [2.3–27.3]
											Any substance disorder	5.6% vs 15.2% OR 3.0 [1.4–6.5]
Secnik et al [42]	2005	US	4,504	64.3%	18≤	ICD-9	ICD-9	Case-control study	General sample–HPM database	NA, (2,252/2,252)	Drug or alcohol abuse	1.87% vs 5.11%
Clinical sample												
Valsecchi et al [52]	2021	Italy	590	47.2%	18–70	ASRS-S v1.1 (+) and DIVA 2.0 both(+)	M.I.N.I Plus	cross-sectional observational study	Psychiatric outpatients	5.12% (590/44)	Substance abuse, lifetime	15.1% vs 29.6% χ2 = 6.34
											Substance abuse, actual	6.6% vs 25.0% χ2 = 19.06
											Substance use, lifetime	30.5% vs 54.6% χ2 = 10.84 .001
											Substance use, actual	8.3% vs 29.6% χ2 = 20.93 .000
Woon and Zakaria [45]	2019	Malaysia	120	94.2%	18–65	CAADID	M.I.N.I	Cross-sectional study	Psychiatric sample -Forensic ward inpatient	15.8% (101/19)	Alcohol abuse	2 (2.0%) vs 3 (15.8%) 0.028
Roncero et al [41]	2019	Spain	726	72.5%	18≤	ASRS (14≤)	DSM-IV-TR	Cross-sectional study	Psychiatric patients–treatment seeking AUD patients	21.1% (573/153)	Cannabis dependence	18% vs 30.9% χ^2^ = 12.3 OR 2.04 [1.36, 3.06]
											Cocaine dependence	24.6% vs 53.3% χ^2^ = 46.5 OR 3.5 [2.41, 5.07]
											Smoking dependence	72.2% vs 82.9% χ^2^ = 6.9 OR 1.86 [1.16, 2.98]
Reyes et al [48]	2019	US	472	64.6%	18–80	PRISM	PRISM	Cross-sectional study	Psychiatric sample–inpatient & outpatient with DSM-IV-TR AUD diagnosis	6.36% (30/442)	Cannabis abuse, Current	41 (9.3%) vs 8 (26.7%)
											Amphetamine abuse, current	17 (3.9%) vs 4 (13.3%)
											Opioid abuse, current	9 (2.0%) vs 3 (10.0%)
Leung and Chan [37]	2017	Hong Kong	254	28.7%	18–64	ASRS-v1.1 ≥17 & SDS ≥5(Screening) + DIVA 2.0 (Diagnosis)	DSM-5	cross-sectional cohort study	Psychiatric sample–clinical outpatients	19.3% (49/205)	Chronic alcohol use	(2.4% vs 8.2%)
											Problematic gambling	(1% vs 2%)
											Active substance use	(3.9% vs. 8.2%)
Retz et al [12]	2016	Germany	163	86.5%	Mean age 40.2 (SD 9.4)	DSM-5 & WURS-k ≥ 30	ICD-10	Cross-sectional study	Psychiatric sample–GD dx according to ICD-10	25.2% (41-current ADHD/122)	Substance use disorders	4’50 (30.7%) vs 19 (46.3%) χ2 = 6.50
Fatséas et al [33]	2016	France	217	66.4%	18–65	CAADID	DSM-IV for SUD SCID-II for BPD M.I.N.I for others	Cross-sectional cohort study	Psychiatric sample–addiction outpatient clinic	23.0% (50/167)	Cannabis dependence	25.9% vs 58.0%
Duran et al [32]	2014	Turkey	246	NA	18–60	WURS score >36 & Turgay’s Adult ADD/ADHD Evaluation Scale	SCID-I-CV, SCID-II	Cross-sectional study	Psychiatric sample - outpatient visit patient	15.9% (39/207)	Other Substance Abuse	12 (5.8%) vs 7 (18.0%) χ2 = 28.81
Perugi et al [40]	2013	Italy	96	59.4%	18–65	ASRS v 1.1 (+), & prior age 7 with ADHD sx	DSM-IV	Cross-sectional observation study	Psychiatric sample- Bipolar I, II disorder diagnosed	19.8% (19/77)	Alcohol	7 (9.1%) vs 5 (26.3%) χ2 = 4.1
											Substance use disorder	14 (18.2%) vs 8 (42.1%) χ2 = 7.1
Incarcerated sample												
Moore et al [47]	2016	Australia	88	76%	18–72	ASRS-S (+) & M.I.N.I plus (+)	M.I.N.I plus, PDQ-4, SCID-II	Cross-sectional study	Incarcerated sample	17.0% (15/73)	Benzodiazepine dependence (lifetime)	13.7% vs 53.3 OR = 5.30 ([1.30, 21.72])
Konstenius et al [23]	2015	Sweden	96	0%	Mean age 39.7	ASRS-S(+) & CAADID	M.I.N.I	Cross-sectional study	Incarcerated sample- only women	29% (16/40)	Drug dependence	58% vs 100%
Capuzzi et al [24]	2022	Italy	108	100%	18–65	WURS-25 & ASRS V1.1	DSM-5	Cross-sectional study	Incarcerated sample- only men	32.4% (35/73)	Cannabis use disorder	40 (54.8%) vs 25 (71.4%)
											Cocaine use disorder	47 (64.4%) vs 32 (91.4%)

OR: Odd Ratio, PR: Prevalence Ratio, NA: Not available (not identified in article)

SUD: Substance Use Disorder, AUD: Alcohol use disorder, GD: Gambling disorder CIDI: Composite International Diagnostic Interview, CIDI-SAM: CIDI-Substance Abuse Module, SCID-I: Structured Clinical Interview for DSM-IV Axis I Disorders, WHO-CIDI: World Health Organization version of the Composite International Diagnostic Interview, M.I.N.I: Mini-International Neuropsychiatric Interview, CAADID: Conner’s Adult ADHD Diagnostic Interview for DSM-IV, ASRS-S: Adult Self-Report Scale-Screener, ASRS: Adult Self-Report Scale, BADDS: Brown attention deficit disorder scale, PRISM: Psychiatric research interview for substance and mental disorders SDS: Sheehan Disability Scale, DIVA: Diagnostic Interview for ADHD in Adults, WURS: Wender Utah Rating Scale, WURS-k: German short form of the Wender Utah rating scale, PDQ-4: Personality disorder diagnostic questionnaire for the DSM-IV, SCID-I-CV: Structured Clinical Interview for DSM-IV Axis I Disorders, Clinician Version

### Prevalence of personality disorders

Fourteen studies provided data comparing the prevalence of personality disorders (including borderline personality disorder and antisocial personality disorder) between ADHD and non-ADHD individuals [12, 16, 23, 24, 30, 33, 34, 38, 41–44, 47, 53]. In general populations, the prevalence of any personality disorders in the non-ADHD group was estimated at 0% [42] to 3.9% [16] compared to 0.31% [42] to 33.8% [16] in the ADHD group. In clinical populations, the prevalence of any personality disorder in the non-ADHD group was estimated at 6.6% [33] to 34.4% [12] compared to 21.9% [34] to 65.95% [12] in the ADHD group. Two studies of incarcerated populations showed differences in the prevalence of borderline personality disorder and antisocial personality disorder. The prevalence of antisocial personality disorder was higher in the ADHD group in both studies [23, 47], and the prevalence of borderline personality disorder was higher in one study [47]. Detailed information from each study is summarized in Table 4.

**Table 4:** Studies comparing the prevalence of personality disorder between non-ADHD and ADHD subjects.

Author	Year	Country	N	Male; %	Age	Assessment of ADHD	Assessment of comorbid psychiatric disorder	Design	Sample	Prev.of ADHD(%) (non-ADHD/ADHD)	Findings comparing non-ADHD and ADHD and prevalence of comorbid psychiatric disorders	non-ADHD, n (%) vs ADHD, n (%)
General sample												
Solberg et al [43]	2018	Norway	1,701,206	51.2%	18≤	ADHD medication at adult or ADHD diagnosis registered	ICD-10	Cross-sectional study	General sample	2.4% (40,103/1,661,103)	Personality disorder	Women 14,079 (1.7%) vs 2,428 (13.6%) Men 8909 (1.1%) vs 2030 (9.1%)
Yoshimasu et al [16]	2016	US	5,718	NA	Mean age ADHD 30.2 (SD 1.9) Non-ADHD controls 30.2 (SD 2.0)	Childhood-identified ADHD with M.I.N.I (+)	M.I.N.I.	Case-control study	General population– Birth cohort sample	NA, (68/335)	Antisocial personality disorder	13 (3.9%) vs 23 (33.8%) OR adj 12.2 [5.3, 27.9]
Miller et al [38]	2007	US	363	51.0%	18–37	K–SADS & structured interview	SCID–I, SCID-II	Case-control study	General sample- Recruited ADHD vs control group	NA, (152/211)	ASPD (Any ADHD)	χ2 = 7.32
Secnik et al [42]	2005	US	4504	64.3%	18≤	ICD-9	ICD-9	Case-control study	General sample–HPM database	NA, (2,252/2,252)	Antisocial disorder Oppositional disorder	0% vs 0.31% 0.04% vs 0.53%
Clinical sample												
Sánchez-García et al [53]	2021,	Puerto-rico, Hungary, Australia	402	79.6%	18–65	CAADID	M.I.N.I Plus	Cross-sectional study	Psychiatric inpatients & outpatients with SUD	35.75% (257/143)	ASPD BPD	25.41% vs 53.90% OR 3.26 [2.09, 5.08] 20.82% vs 57.45% OR 5.48 [3.40, 8.83]
Roncero et al [41]	2019	Spain	726	72.5%	18≤	ASRS (14≤)	DSM-IV-TR	Cross-sectional study	Psychiatric patients–treatment seeking AUD patients	21.1% (573/153)	Any personality disorder	14.8% vs 37.4% χ^2^ = 38.17 .0001 OR 3.45 [2.29, 5.17]
Gorlin et al [34]	2016,	US	1,134	42%	Mean age 39.7 (SD 14.4)	DSM-IV based semi-structured clinical interview	SCID	Cross-sectional study	Psychiatric sample-clinical outpatient	18.0% (204/903)	Borderline personality disorder	7.6% vs 21.9% OR = 3.11 (2.02–4.76)
Retz et al [12]	2016	Germany	163	86.5%	Mean age 40.2 (SD 9.4)	DSM-5 & WURS-k ≥ 30	ICD-10	Cross-sectional study	Psychiatric sample–GD dx according to ICD-10	25.2% (41-current ADHD/122)	Personality disorders Cluster B	56 (34.4%) vs 27 (65.9%) χ2 = 26.84 11 (6.7%) vs 7 (17.1%) χ2 = 30.49
Fatséas et al [33]	2016	France	217	66.4%	18–65	CAADID	DSM-IV for SUD SCID-II for BPD M.I.N.I for others	Cross-sectional cohort study	Psychiatric sample–addiction outpatient clinic	23.0% (50/167)	Antisocial personality disorder Borderline personality disorder	6.6% vs 26.0% 13.0% vs 34.7%
van Emmerik-van Oortmerssen et al [44]	2014	Australia, Belgium, France, Hungary, Netherlands, Norway, Spain, Sweden, Switzerland, US (IASP study) (37)			18–65	CAADID	M.I.N.I Plus SCID-II	Cross-sectional study	Psychiatric sample- treatment-seeking SUD patients	13.9% (168/1,037)	ASPD BPD	17.0% vs 51.8% OR 2.8 [1.8–4.2] -alcohol 8.2% vs 34.5% OR 7.0 [3.1–15.6] -drugs 16.7% vs 29.0% OR 3.4 [1.8–6.4]
Olsson et al [30]	2022	Sweden	804	67.3%	18≤	ICD-10 or prescription of ADHD medication	ICD-10	Cross-sectional study	Psychiatric sample–psychiatric emergency patient with NSSI	11.6% (711/93)	Personality disorder	142 (20%) vs 28 (30), χ2 = 5.07
Incarcerated sample												
Moore et al [47]	2016	Australia	88	76%	18–72	ASRS-S (+) & MINI plus (+)	M.I.N.I plus, PDQ-4, SCID-II	Cross-sectional study	Incarcerated sample	17.0% (15/73)	BPD ASPD	13.7% vs 60.0% OR = 7.34 ([1.72, 31.37]) 27.4% vs 93.3% OR = 26.00 ([2.58, 262.30])
Konstenius et al [23]	2015	Sweden	96	0%	Mean age 39.7	ASRS-S (+) & CAADID	M.I.N.I	Cross-sectional study	Incarcerated sample- only women	29% (16/40)	ASPD	30% vs 81%
Capuzzi et al [24]	2022	Italy	108	100%	18–65	WURS-25 & ASRS V1.1	DSM-5	Cross-sectional study	Incarcerated sample- only men	32.4% (35/73)	Personality disorders	26 (35.6%) vs 21 (60.0%)

OR: Odd Ratio, NA: Not available (not identified in article)

SUD: Substance Use Disorder, AUD: Alcohol use disorder, GD: Gambling disorder BPD: Borderline Personality Disorder, ASPD: Antisocial personality disorder

SCID: Structured Clinical Interview for DSM-IV, M.I.N.I: Mini-International Neuropsychiatric Interview, ASRS-S: Adult Self-Report Scale-Screener, ASRS: Adult Self-Report Scale, CAADID: Conner’s Adult ADHD Diagnostic Interview for DSM-IV, WURS-k: German short form of the Wender Utah rating scale, K-SADS: Kiddie Schedule for Affective Disorders and Schizophrenia, PDQ-4: Personality disorder diagnostic questionnaire for the DSM-IV

HPM: Health and Productivity Management

ISAP: International ADHD in Substance use disorder Prevalence

## Discussion

In our systematic review, we included 32 studies conducted over 16 years dealing with the prevalence of psychiatric comorbidities between adults with and without ADHD. To our knowledge, this is the first systematic review comparing the prevalence of comprehensive comorbid psychiatric disorders between adults with and without ADHD and including both general and clinical populations. Articles published from 2006 to 2022 were included in this review. This might because the interest in adult ADHD had increased based on the results of longitudinal follow-up studies on children and adolescents with ADHD [54, 55].

The studies included in this review most commonly used ASRS as a diagnostic tool for adult ADHD (12 of 32), followed by CADDID and WURS. As a self-report scale, due to its simplicity and cost-effectiveness, ASRS can be preferred by ADHD investigators. However, the variability in the evaluation tools for adult ADHD is thought to be due to the lack of established diagnostic criteria [56]. Many psychiatric disorders are diagnosed based on observation of an assessor and complaint of a patient. The lack of standardized diagnostic tools can lead to over-diagnosis or under-diagnosis of psychiatric disorders [57, 58] and can cause inconsistency in diagnosis, which complicates comparison studies [59, 60]. Heterogeneity of diagnostic tools observed in this study not only prevented meta-analysis, but also might affect the variability in the reported prevalence of adult ADHD.

In addition, the prevalence of ADHD in adults varied from 1.1% [39] to 8.8% [50] in general population samples and from 5.12% [52] to 35.75% [53] in psychiatric population samples. A previous systematic review of ADHD prevalence in and adult psychiatric population shows a similar range, from 6.9% to 38.75% [61]. In this previous study [61], the authors assumed that this variation might be due to the diversity of diagnostic methods and the inclusion and exclusion criteria in the studies. Similarly, in our study, the aforementioned variability of diagnostic methodologies for ADHD might have affected this various range of prevalence. In a general population, the estimated mean prevalence rate of ADHD in adults was 2.8% in a previous study [15]. Except in two studies included in our review, targeting special populations of army soldiers [51] and twins of Sweden [50], which had higher than estimated prevalence, the range of ADHD prevalence in the general population was 1.1% to 2.9% in our study, similar to that previously observed [11, 56].

The most frequent comorbid psychiatric disorder in the ADHD group was SUD, its prevalence ranging from 2.3% and 41.2% in the general population and between 10.0% and 82.9% in the clinical population; two of three studies showed significant prevalence difference between ADHD and non-ADHD subjects. This finding correlates with a previous meta-analysis that reported that almost one out of every four adolescent and adult patients with SUD presents with ADHD [62, 63], which supports SUD as one of the most frequent comorbid psychiatric conditions in adult ADHD. There are some theoretical opinions of shared key characteristics and pathophysiology between ADHD and SUD, like dopaminergic dysregulation of motivational and reward systems, or reduced frontal function including executive functions and response inhibition [64, 65]. In addition, considering that childhood ADHD is a prominent risk factor for substance misuse and development of SUD due to the most frequent comorbidities in childhood ADHD, like conduct disorder or oppositional defiant disorder [66], untreated and preserved ADHD in adults might have influenced the cross-sectional difference of prevalence rate between ADHD and non- ADHD patients.

Mood disorders, including depressive disorders and bipolar disorders, were also frequently observed comorbid psychiatric disorders in ADHD subjects compared to non-ADHD subjects. The estimated prevalence of depressive disorders in the ADHD group ranged from 8.6% to 55% in the general population and 15.4% to 39.7% in the clinical population. Also, the prevalence of bipolar disorder in the ADHD group was estimated at 4.45% to 35.3% in the general population and 7.4% to 80.8% in the clinical population. For depression, previous studies have also shown a higher prevalence of depressive disorders in young adults with ADHD compared to non-ADHD subjects as well as higher risk of suicidal behavior [67, 68]. This can be explained by a previous cross-sectional study showing the association between ADHD symptoms and depressive symptoms in young adults as identified by low hedonic responsivity [69]. In addition, according to biologic aspects of depression and ADHD, the two disorders might share similar pathophysiologic regions of the brain including decreased activity in the prefrontal [70, 71], amygdala, and hippocampus regions [72–74]. Furthermore, 10 studies showed a higher prevalence of bipolar disorder, including current hypomania diagnosed by SCID-II, in ADHD subjects than in non-ADHD subjects. Considering that the worldwide prevalence rate of bipolar disorder is estimated as 1–3% [75, 76], which was similar to that of the non-ADHD general population in our study, the prevalence of bipolar disorder in the ADHD group was greater than 3% in all 10 studies. This finding correlated with previous studies reporting reciprocal high comorbidity rates between ADHD and bipolar disorder, which suggests possible shared genetic effects or diagnostic overlap between the two disorders [77].

In anxiety disorder, almost two of three studies showed a higher prevalence in the ADHD group than the non-ADHD group. The prevalence rate in the ADHD group was estimated to range from 4.3% to 47.1% in the general population and from 3.9% to 84% in the clinical population. Only one study of a clinical population dealing with psychiatric outpatients in the US [34] showed a higher prevalence of adjustment disorder in the non-ADHD group. These findings correlate with previous studies that revealed a high prevalence of anxiety in the adult ADHD population [78, 79]. ADHD seems to show different characteristics from anxiety, namely fearlessness and impulsivity. Therefore, various theories have been suggested to explain this phenomenon using developmental or biologic aspects in children and adolescents [80]. Similarly, in adults, as far as we know, the two disorders have been related to several common neuroanatomical regions like the dorsolateral prefrontal cortex or the anterior cingulate cortex, which are critically involved with the executive function control network [10]. In addition, considering a previous study about increased risk of accidents in ADHD over the lifespan [81], traumatic events might have influenced the higher prevalence of anxiety disorders. From a developmental viewpoint, as in depressive disorders, this frequently higher prevalence of anxiety disorder might represent the social and relational difficulties induced by ADHD.

The estimated prevalence of personality disorders in the ADHD group was ranged from 0.31% to 33.8% in the general population and from 21.9% to 65.95% in the clinical population. Previous studies have reported that personality disorders, mostly cluster B or C personality disorders, are present in almost 50% of adults with ADHD [82]. The association between ADHD and personality disorders might be mediated by the symptomatic dimensions of ADHD such as emotional dysregulation and oppositional symptoms [83]. In our review, most studies showed a higher prevalence of cluster B personality disorders in ADHD than in non-ADHD groups. Specifically, in the clinical population, more than 20% of adult ADHD subjects were estimated to have comorbid cluster B personality disorder including borderline personality disorder and antisocial personality disorder. Additionally, most clinical population studies included patients diagnosed with substance use disorders, which correlates with previous observational studies of young male adults with ADHD that revealed associations of antisocial personality disorder with ADHD [84].

### Limitations

There are several limiting factors in this review. As mentioned previously, there is significant heterogeneity across studies diagnosing both ADHD and comorbid psychiatric disorders. This prohibited meta-analysis. Furthermore, except for two international studies [44, 53], the included studies were conducted in high-income regions like Europe or North America. In a previous epidemiologic study investigating cross-national ADHD prevalence in adults [15], the prevalence differed by country income, with higher rates observed in higher-income countries. This difference in prevalence among countries affects the degree of interest in the disease, which might be why the included studies were mainly conducted in Europe and North America. In addition, we did not differentiate patients according to ADHD or comorbid psychiatric disorder treatment status, which might also have affected the prevalence of comorbid psychiatric disorders. Of the included studies, most explored the prevalence cross-sectionally, so we could not infer a correlation or antecedent relationship between ADHD and comorbidities. Only limited estimates of the associations between ADHD and comorbidities can be provided by our review at the study level. Additionally, we did not assess the risk of bias in each study. To include as many studies as possible to reflect broad and various studies of different countries, populations, and comorbid psychiatric disorders, we omitted the risk of bias assessment. Also, there was no pre-registration for our systematic literature review. These points are limitations to this study.

## Conclusion

In conclusion, our findings indicate a higher prevalence of comorbid psychiatric disorders in ADHD subjects compared to non-ADHD subjects, whether they were previously diagnosed with other psychiatric disorders or not. Furthermore, our results suggest a complex association between the multiple comorbidities of ADHD. Given that ADHD is often unrecognized and under-diagnosed in adults, screening for ADHD might be beneficial for patients presenting multiple psychopathologies including substance abuse, mood disorders, and anxiety disorders. In the future, research on standardization of ADHD diagnosis in adults and its comorbid psychopathologies will be required to distinguish adult ADHD from comorbid psychiatric disorders, and to enable comparison among study conditions. This standardization will aid in differential diagnosis and allow provision of earlier treatment in adult ADHD. In addition, research on the neurobiological and developmental bases of ADHD and its comorbid psychiatric disorders should continue to improve the understanding of the connectivity and associations between various comorbid psychiatric disorders and ADHD in adults.

## Supporting information

S1 Table PRISMA checklist. (DOCX)

S1 Text Article search strategy. (DOCX)
