# Supplementary material for: The prevalence of psychiatric comorbidities in adult ADHD compared with non-ADHD populations: A systematic literature review
Source: PLoS One. 2022 Nov 4;17(11):e0277175. doi: 10.1371/journal.pone.0277175 (PMC9635752; doi:10.1371/journal.pone.0277175)
Supplement: S1 Text — (DOCX) [file pone.0277175.s002.docx]

**Supplement 2. Article search strategy**

**Pubmed**

Total : 503

(ADHD OR ADD OR attention deficit) AND (adult) AND (comorbidity OR comorbid) AND (prevalence) in abstract or titles

487 results on 22/July/2021

16 more results from 22/July/2021 to 01/August/2022

**EMBASE**

Total : 464

(adhd OR add OR attention deficit) AND adult AND (comorbidity OR comorbid) AND prevalence *.mp.

358 results on 22/July/2021

106 more results from 22/July/2021 to 01/August/2022

**PsycINFO**

Total : 661

(ADHD OR ADD OR attention deficit) AND (adult) AND (comorbidity OR comorbid) AND (prevalence)

only peer reviewed journal

620 results on 22/July/2021

41 more results from 22/July/2021 to 01/August/2022

**PsycNET**

Total : 11

11 results on 22/July/2021

No more updates after 22/July/2021

**Google Scholar**

Total : 129

allintitle: adult ADHD comorbid OR comorbidity

113 results on 22/July/2021

16 more results from 22/July/2021 to 01/August/2022
