# Supplementary material for: The prevalence of psychiatric comorbidities in adult ADHD compared with non-ADHD populations: A systematic literature review
Source: PLoS One. 2022 Nov 4;17(11):e0277175. doi: 10.1371/journal.pone.0277175 (PMC9635752; doi:10.1371/journal.pone.0277175)
Supplement: Attachment — Submitted filename: PONE-D-22-17093.pdf [file pone.0277175.s003.pdf]

PLOS ONE

The Prevalence of Psychiatric Comorbidities in Adult ADHD Compared With Non-ADHD Populations : A Systematic Literature Review

--Manuscript Draft--

**Manuscript Number:**	PONE-D-22-17093
**Article Type:**	Research Article
**Full Title:**	The Prevalence of Psychiatric Comorbidities in Adult ADHD Compared With Non-ADHD Populations : A Systematic Literature Review
**Short Title:**	The Prevalence of Psychiatric Comorbidities in Adult ADHD
**Corresponding Author:**	Won-Myong Bahk<br>Catholic University of Korea College of Medicine: Catholic University of Korea School of Medicine<br>KOREA, REPUBLIC OF
**Keywords:**	Attention Deficit Hyperactivity Disorder (ADHD); Adults; Comorbidity; Prevalence; Mood disorders; Anxiety disorders; Substance-related disorders; Personality disorders
**Abstract:**	Comorbid psychiatric disorders in adults with ADHD are important because these comorbidities might complicate the diagnosis of ADHD and also worsen the prognosis. However, the prevalence of comorbid psychiatric disorders in adult ADHD varies according to the diagnostic tools used and the characteristics of target populations. The purpose of this review was to describe the prevalence of comorbid psychiatric disorders in adults with ADHD compared with adults without ADHD. Thirty studies published before July 2021 were identified and classified according to diagnosis of other psychiatric disorder in those with ADHD. The most frequent comorbid psychiatric disorder in the ADHD group was substance use disorder (SUD), followed by mood disorders, anxiety disorders, and personality disorders. The prevalence of these four disorders was higher in the ADHD group, whether or not subjects were diagnosed with other psychiatric disorders. In addition, the diversity of ADHD diagnostic tools was observed. This also might have affected the variability in prevalence of comorbidities. Standardization of ADHD diagnostic tools is necessary in the future.
**Order of Authors:**	Won-Seok Choi
	Young Sup Woo
	Sheng-Min Wang
	Hyun Kook Lim
	Won-Myong Bahk
**Additional Information:**	
**Question**	**Response**
**Financial Disclosure**<br><br>Enter a financial disclosure statement that describes the sources of funding for the work included in this submission. Review the submission guidelines for detailed requirements. View published research articles from *PLOS ONE* for specific examples.<br><br>This statement is required for submission and **will appear in the published article** if the submission is accepted. Please make	The author(s) received no specific funding for this work.

Powered by Editorial Manager® and ProduXion Manager® from Aries Systems Corporation

sure it is accurate.<br><br>**Unfunded studies**<br>Enter: *The author(s) received no specific funding for this work.*<br><br>**Funded studies**<br>Enter a statement with the following details:<br>• Initials of the authors who received each award<br>• Grant numbers awarded to each author<br>• The full name of each funder<br>• URL of each funder website<br>• Did the sponsors or funders play any role in the study design, data collection and analysis, decision to publish, or preparation of the manuscript?<br>• **NO** - Include this sentence at the end of your statement: *The funders had no role in study design, data collection and analysis, decision to publish, or preparation of the manuscript.*<br>• **YES** - Specify the role(s) played.<br><br>* typeset	
**Competing Interests**<br><br>Use the instructions below to enter a competing interest statement for this submission. On behalf of all authors, disclose any competing interests that could be perceived to bias this work—acknowledging all financial support and any other relevant financial or non-financial competing interests.<br><br>This statement is required for submission and **will appear in the published article** if the submission is accepted. Please make sure it is accurate and that any funding sources listed in your Funding Information later in the submission form are also declared in your Financial Disclosure statement.<br><br>View published research articles from *PLOS ONE* for specific examples.	The authors have declared that no competing interests exist.

**NO authors have competing interests**<br><br>Enter: *The authors have declared that no competing interests exist.*<br><br>**Authors with competing interests**<br><br>Enter competing interest details beginning with this statement:<br><br>*I have read the journal's policy and the authors of this manuscript have the following competing interests: [insert competing interests here]*<br><br>* typeset	
**Ethics Statement**<br><br>Enter an ethics statement for this submission. This statement is required if the study involved:<br><br>• Human participants<br>• Human specimens or tissue<br>• Vertebrate animals or cephalopods<br>• Vertebrate embryos or tissues<br>• Field research<br><br>Write "N/A" if the submission does not require an ethics statement.<br><br>General guidance is provided below. Consult the submission guidelines for detailed instructions. **Make sure that all information entered here is included in the Methods section of the manuscript.**	N/A

**Format for specific study types**<br><br>**Human Subject Research (involving human participants and/or tissue)**<br>• Give the name of the institutional review board or ethics committee that approved the study<br>• Include the approval number and/or a statement indicating approval of this research<br>• Indicate the form of consent obtained (written/oral) or the reason that consent was not obtained (e.g. the data were analyzed anonymously)<br><br>**Animal Research (involving vertebrate animals, embryos or tissues)**<br>• Provide the name of the Institutional Animal Care and Use Committee (IACUC) or other relevant ethics board that reviewed the study protocol, and indicate whether they approved this research or granted a formal waiver of ethical approval<br>• Include an approval number if one was obtained<br>• If the study involved *non-human primates*, add *additional details* about animal welfare and steps taken to ameliorate suffering<br>• If anesthesia, euthanasia, or any kind of animal sacrifice is part of the study, include briefly which substances and/or methods were applied<br><br>**Field Research**<br><br>Include the following details if this study involves the collection of plant, animal, or other materials from a natural setting:<br>• Field permit number<br>• Name of the institution or relevant body that granted permission	
**Data Availability**<br><br>Authors are required to make all data underlying the findings described fully available, without restriction, and from the time of publication. PLOS allows rare exceptions to address legal and ethical concerns. See the PLOS Data Policy and FAQ for detailed information.	Yes - all data are fully available without restriction

Powered by Editorial Manager® and ProduXion Manager® from Aries Systems Corporation

A Data Availability Statement describing where the data can be found is required at submission. Your answers to this question constitute the Data Availability Statement and **will be published in the article**, if accepted.<br><br>**Important:** Stating 'data available on request from the author' is not sufficient. If your data are only available upon request, select 'No' for the first question and explain your exceptional situation in the text box.<br><br>Do the authors confirm that all data underlying the findings described in their manuscript are fully available without restriction?	
**Describe where the data may be found in full sentences. If you are copying our sample text, replace any instances of XXX with the appropriate details.**<br><br>• If the data are **held or will be held in a public repository**, include URLs, accession numbers or DOIs. If this information will only be available after acceptance, indicate this by ticking the box below. For example: *All XXX files are available from the XXX database (accession number(s) XXX, XXX.).*<br>• If the data are all contained **within the manuscript and/or Supporting Information files**, enter the following: *All relevant data are within the manuscript and its Supporting Information files.*<br>• If neither of these applies but you are able to provide **details of access elsewhere**, with or without limitations, please do so. For example:<br><br>*Data cannot be shared publicly because of [XXX]. Data are available from the XXX Institutional Data Access / Ethics Committee (contact via XXX) for researchers who meet the criteria for access to confidential data.*<br><br>*The data underlying the results presented in the study are available from (include the name of the third party*	All relevant data are within the manuscript and its Supporting Information files.

and contact information or URL).<br>• This text is appropriate if the data are owned by a third party and authors do not have permission to share the data.<br><br>* typeset	
Additional data availability information:	

Powered by Editorial Manager® and ProduXion Manager® from Aries Systems Corporation

Manuscript
Click here to access/download;Manuscript;Manuscript_Table.docx

The Prevalence of Psychiatric Comorbidities in Adult ADHD Compared With Non-ADHD Populations : A Systematic Literature Review

Won-Seok Choi[1¶], Young Sup Woo[2¶], Sheng-Min Wang[2], Hyun Kook Lim[2], Won-Myong Bahk[2*]

[1]Department of Psychiatry, Hallym University Sacred Heart Hospital, Anyang, Korea

[2]Department of Psychiatry, College of Medicine, The Catholic University of Korea, Seoul, Korea

*Corresponding Author

wmbahk@catholic.ac.kr (WMB)

¶ These authors contributed equally to this study.

Abstract

Comorbid psychiatric disorders in adults with ADHD are important because these comorbidities might complicate the diagnosis of ADHD and also worsen the prognosis. However, the prevalence of comorbid psychiatric disorders in adult ADHD varies according to

the diagnostic tools used and the characteristics of target populations. The purpose of this review was to describe the prevalence of comorbid psychiatric disorders in adults with ADHD compared with adults without ADHD. Thirty studies published before July 2021 were identified and classified according to diagnosis of other psychiatric disorder in those with ADHD. The most frequent comorbid psychiatric disorder in the ADHD group was substance use disorder (SUD), followed by mood disorders, anxiety disorders, and personality disorders. The prevalence of these four disorders was higher in the ADHD group, whether or not subjects were diagnosed with other psychiatric disorders. In addition, the diversity of ADHD diagnostic tools was observed. This also might have affected the variability in prevalence of comorbidities. Standardization of ADHD diagnostic tools is necessary in the future.

Introduction

ADHD(attention-deficit/hyperactivity disorder) is a common psychiatric disorder presenting persistent inattention and/or hyperactivity/impulsiveness (1), which is associated with considerable problems in personal, social, and occupational areas (2). While ADHD is well studied in children, it is recently being studied in adults as well. According to a previous meta-analysis, 65% of children who were diagnosed with ADHD have persistent ADHD symptoms in adulthood (3). In addition, the prevalence of ADHD in adults is known to reach 2.5% (4), which is moderate compared to its prevalence in children, which is about 5% (5).

Although comorbid psychiatric disorders are common in both adults and children, the comorbidity rate is higher in adults; as many as 80% of adults with ADHD are reported to have at least one comorbid psychiatric disorder (6, 7, 8). In clinical adult ADHD samples, substance use disorder (SUD), mood disorder, anxiety disorder, and antisocial personality disorder

(ASPD) are the most common comorbid disorders (9, 10), and these mental disorders can adversely affect patient prognosis. Furthermore, research revealed that comorbid psychiatric disorders cause considerable functional impairment in individuals with ADHD and place a great burden on society (11).

For this reason, several cross-sectional studies have been conducted on various populations including clinical and general settings over 30 years to evaluate the prevalence of comorbid psychiatric conditions in adults with ADHD. However, the prevalence of comorbid psychiatric disorders varied according to characteristics of the subjects, including country, race, gender, and other socioeconomic characteristics as well as the screening or diagnostic tools applied (10, 12). Moreover, since ADHD has been recognized in adults, diagnostic tools for adult ADHD and its comorbid disorders have changed over time (13), and the interest in clinical diagnoses and optimal treatments in adults with ADHD has also increased (14). These factors might have contributed to the divergent prevalence rate of ADHD and comorbid disorders in adults.

However, to the best of our knowledge, despite the high incident rates of psychiatric disorders documented in previous studies in the adult ADHD subjects (10, 11, 15, 16) and their importance in the clinical field, no systematic literature review has specifically compared the degree of comorbid psychiatric disorders between adults with and without ADHD. Thus, the aim of our study was to ascertain the difference in the prevalence rates of comorbid psychiatric disorders between adults with and without ADHD including both clinical and general populations.

Methods

Study search and data sources

The methodology of the present review was followed by Preferred Reporting Items for Systematic Reviews and Meta-Analysis (17) (available as Supporting Information, S1 table.). We searched electronic libraries of PubMed, EMBASE, PsycINFO, PsycNET, and Google Scholar for publications regarding the epidemiology and prevalence rate of comorbidities of adult ADHD published prior to 22 July 2021. The initial search was conducted using the following terms : Prevalence AND (ADHD OR ADD OR Attention Deficit) AND Adult AND (comorbidity OR comorbid) in titles or abstracts.

Study selection

First, articles obtained from the initial search were de-duplicated by EndNote 20. Then, inclusion/exclusion screening was performed based on exclusion criteria of non-relevant articles (e.g., did not focus on adult patients or did not include psychiatric comorbidity data), non-English articles, full text not available, abstract-only papers, articles that were not peer-reviewed, and reviews or meta-analyses. The initial inclusion/exclusion review was completed based on titles and abstracts, and if the relevance of the article was unclear, a full-text review was performed. After this initial process, the full texts of all included articles were retrieved to evaluate our detailed eligibility criteria. Articles were included in the study if they 1) used samples of adult populations aged 18 years or older, 2) defined clear ADHD and non-ADHD groups by clinically diagnoses or using any diagnostic criteria (e.g., DSM (Diagnostic and Statistical Manual of Mental Disorders)) or tools for screening/diagnosing ADHD in adults (e.g., ASRS (Adult ADHD Self-report Scale)), 3) defined the prevalence rate of comorbid psychiatric disorders using any diagnostic tools for each psychiatric disorder (e.g. SCID (Structured Clinical Interview for DSM-IV)), and 4) directly compared ADHD and non-ADHD groups using statistical analysis.

Data collection process

Microsoft Excel was used to develop a data extraction sheet, and the full-text articles were reviewed randomly by two researchers (WSC, YSW) who also conducted the initial data search. The extracted data were reviewed for consistency, and any queries that arose were resolved by discussion among the researchers. The lead author decided whether to include/exclude data by reviewing the specific articles.

Measurements

Because there are various methods for diagnosing ADHD and psychiatric disorders in adults, we extracted the following variables from the articles ultimately included: 1) data describing the study characteristics, such as year of publication, country, or study design; 2) data describing the target population, like sample size, age range or mean age/SD, or gender composition; 3) diagnostic tools for adult ADHD and comorbid psychiatric disorders, whether clinical diagnosis was performed, and the diagnostic criteria for ADHD/psychiatric comorbidity; 4) study results including the prevalence rate of ADHD in the target population, prevalence rate of each psychiatric comorbidity in each ADHD and non-ADHD group, and any statistically significant comparable variables including odd ratios(ORs) with 95% confidence intervals or chi-square (χ^2) test variables.

Classification of studies

Based on several studies targeting nation-wide psychiatric comorbidities (18-20), assuming that the prevalence of comorbid psychiatric disorders is higher in groups of psychiatric patients, we decided to divide the study populations into general population group studies and clinical

group studies. A clinical group study was defined as one in which the study population included patients who had previously been clinically diagnosed with any psychiatric disorder or had visited/been admitted either voluntarily or involuntarily to a hospital for treatment.

In addition, considering the specificity that the prevalence of ADHD among incarcerated people was five to 10 times higher than that of the general population (21), and that the prevalence of comorbid psychiatric disorders among inmates was higher than that of the general population (22), we classified data of incarcerated patients separately from other population groups.

Results

In total, 1589 articles were identified by the search method described above, and 314 duplicates were removed. After the duplicates excluded, an additional 1017 articles were excluded by screening titles and abstracts. The remaining 258 articles were read in full and included in the analysis if they met the inclusion criteria of our study. Based on our study criteria, 228 articles were excluded for reasons noted in Fig 1. Thus, 30 studies of comparing the prevalence rates of comorbid psychiatric disorders between ADHD and non-ADHD adult subjects were selected for systematic review.

Of the 30 studies comparing the prevalence of comorbid psychiatric disorder between subjects with and without adult ADHD, according to our classification criteria, 11 studies involved general populations, 17 studies included psychiatric populations, and two studies focused on incarcerated populations. One of the two studies dealing with incarcerated populations involved only female inmates (23).

Prevalence of mood disorders

Eighteen studies provided data comparing the prevalence of mood disorders (including depressive disorders and bipolar disorders) between ADHD patients and non-ADHD individuals (6, 16, 24-39). In the general populations, the prevalence of any depressive disorder in the non-ADHD group was estimated at 1.2% (16) to 12.5% (29), compared to 8.6% (30) to 55% (6) in the ADHD group. In clinical populations, the prevalence of any depressive disorder in the non-ADHD group was estimated at 5.8%(26) to 39.6%(28), compared to 15.4%(26) to 39.7%(38) in the ADHD group. In the general population, the prevalence of any bipolar disorder in the non-ADHD group was estimated at 0.2%(29) to 3.6%(16) compared to 4.48%(36) to 35.3%(16) in the ADHD group. In clinical populations, the prevalence of any bipolar disorder in the non-ADHD group was estimated at 2.0% (31) to 19.5% (34), compared to 7.4% (28) to 80.0.% (24) in the ADHD group. There were no differences reported in the prevalence of mood disorders between ADHD and non-ADHD groups in the incarcerated population studies. Detailed information from each study is summarized in Table 1.

Prevalence of anxiety and related disorders

Sixteen studies provided data comparing the prevalence of anxiety disorders including obsessive-compulsive disorder, somatoform disorders and trauma/stress-related disorders between ADHD patients and non-ADHD individuals (12, 16, 25, 28-30, 32, 33, 35-37, 39-43). In general population, the prevalence of any anxiety disorders in the non-ADHD group was estimated at 0.5% (33) to 9.5% (30) compared to 4.3% (33) to 47.1% (30) in ADHD group. In clinical populations, the prevalence of any anxiety disorders in the non-ADHD group was estimated at 5.4% (40) to 40% (43) compared to 3.9% (28) to 84% (43) in the ADHD group. Only one study of incarcerated populations showed a difference in the prevalence of social phobia between non-ADHD and ADHD individuals (41). Detailed information from each study

is summarized in Table 2.

Prevalence of substance use disorders and gambling disorder

Twenty-one studies provided data comparing the prevalence of substance use disorders (including addiction to alcohol, opioids, stimulants, cannabis, anxiolytics, and nicotine) and gambling disorders between ADHD and non-ADHD individuals (6, 12, 16, 23, 25-27, 29-36, 39, 41, 42, 44-46). In general populations, the prevalence of any substance use disorder in the non-ADHD group was estimated at 0% (6) to 16.6% (33) compared to 2.3% (29) to 41.2% (16) in the ADHD group. In clinical populations, the prevalence of any substance use disorder in the non-ADHD group was estimated to be 2.0% (39) to 72.2% (35) compared to 10.0% (42) to 82.9% (35) in the ADHD group. Two studies compared the prevalence of gambling disorder between ADHD and non-ADHD patients, and there was one study for each general/psychiatric population group, showing a statistically significant difference in prevalence (31, 33). Two studies of incarcerated populations showed differences in the prevalence of benzodiazepine use disorder (41) and drug dependence (23). Detailed information from each study is summarized in Table 3.

Prevalence of personality disorders

Twelve studies provided data comparing the prevalence of personality disorders (including borderline personality disorder and antisocial personality disorder) between ADHD and non-ADHD individuals (12, 16, 23, 27, 28, 32, 35-38, 41, 47). In general populations, the prevalence of any personality disorders in the non-ADHD group was estimated at 0% (36) to 3.9% (16) compared to 0.31% (36) to 33.8% (16) in the ADHD group. In clinical populations, the prevalence of any personality disorder in the non-ADHD group was estimated at 6.6% (27)

to 34.4% (12) compared to 21.9% (28) to 65.95 % (12) in the ADHD group. Two studies of incarcerated populations showed differences in the prevalence of borderline personality disorder and antisocial personality disorder. The prevalence of antisocial personality disorder was higher in the ADHD group in both studies (23, 41), and the prevalence of borderline personality disorder was higher in one study (41). Detailed information from each study is summarized in Table 4.

Table 1. Studies comparing the prevalence of mood disorders between non-ADHD and ADHD subjects									
Author, year, country	N (Male; %)	Age	Assessment of ADHD	Assessment of comorbid psychiatric disorder	Design	Sample	Prev.of ADHD(%) (non-ADHD/ADHD)	Findings comparing non-ADHD and ADHD and prevalence of comorbid psychiatric disorders<br>non-ADHD, n (%) vs ADHD, n (%)	Comment
General sample									
Solberg et al ,2018, Norway (37)	1,701,206 (871,330; 51.2%)	18≤	ADHD medication at adult or ADHD diagnosis registered	ICD-10	Cross-sectional study	General sample	2.4% (40,103/1,661,103)	Bipolar Disorder<br>Women 13,183 (1.6%) vs 2,290 (12.9%)<br>Men 9,009 (1.1%) vs 1,981 (8.9%)<br>Major depressive disorder<br>Women 61,880 (7.6%) vs 5,138 (28.8%)<br>Men 33,733 (4.0%) vs 4,516 (20.3%)	
Chen et al, 2018, Norway (25)	5,551,807 (2,820,859 ; 50.81%)	18-64	ICD-9: 314; ICD-10: F90 diagnosis	ICD	Cross-sectional study	General sample	1.1% (61,129/5,490,678)	Depression<br>PR = 9.01 (8.92–9.10)<br>Bipolar Disorder<br>PR=19.96 (19.48–20.43)	
Hesson and Fowler, 2018, Canada (29)	16,957	20-64	Self-report of ADHD (diagnosed by a health professional)	WHO-CIDI modified for the needs of CCHS-MH	Case-control study	General sample -national mental health survey	2.9% (NA)	12-month<br>Major depressive disorder<br>61 (12.5%) vs 113 (23.3%) χ2 = 59.94<br>Bipolar disorder I 1 (0.2%) vs 20 (4.1%) χ2 = 17.73	
Yoshimasu et al, 2016, US (16)	5,718	Mean age ADHD 30.2 (SD 1.9)	childhood-identified ADHD with	M.I.N.I	Case-control study	General population –Birth	NA, (68/335)	Hypomanic episode - current or past 12 (3.6%) vs 24 (35.3%) OR adj 16.5 [7.2, 37.4]	

		Non-ADHD controls 30.2 (SD 2.0)	M.I.N.I (+)			cohort sample		Dysthymia<br>4 (1.2%) vs 11 (16.2%) OR adj 19.0 [5.4, 66.1]<br>MDD<br>9 (2.7%) vs 19 (27.9%) OR adj 15.2 [6.2, 37.4]	
Park et al, 2011, South Korea (33)	6,081<br>ADHD+ 59.4%<br>ADHD- 50.5%	18-59	ASRS-S v 1.1 (+)	K-CIDI (Korean Ver. of CIDI)	Epidemiological study	General sample	1.1% (69/6,012)	Any mood disorder<br>6.0% vs 27.1% OR 6.44 [3.70–11.19]<br>Major depressive disorder<br>5.5% vs 17.4% OR=4.00[2.10–7.63]<br>Bipolar disorder<br>0.2% vs 8.6% OR 29.94 [10.71–83.66]	
Miller et al, 2007, US (32)	363 (185; 51.0%)	18-37	K–SADS & structured interview	SCID–I, SCID-II	Case-control study	General sample - Recruited ADHD vs control group	NA, (152/211)	Mood disorder<br>NA, χ2= 23.70	
Sobanski et al, 2007, Germany (6)	140 (76; 54.3%)	Mean age ADHD+ 36.8 (SD 9.0)<br>ADHD- 39.8 (SD 10.0)	WURS-K & BADDS	SCID-I	Case-control study	General sample -referred ADHD vs control group	NA, (70/70)	Affective disorders total<br>18 (25.7%) vs 44 (60.7%) χ2= 18.462<br>Major depressive episodes 17 (24.3%) vs 40 (55%) χ2= 15.010	
Kessler et al, 2006, US (30)	3,199	18-44	DIS-IV for childhood	CIDI	Epidemiological study	General sample	2.6% (NA)	Major depressive disorder<br>7.8%vs 8.6% 4.2 OR 2.7[1.5–4.9]	

			pathology & ACDS v 1.2 (ADHD-RS)			– national survey		Dysthymia<br>1.9% vs 12.8% OR 7.5 [3.8–15]<br>Bipolar<br>3.1% vs 19.4% OR 7.5 [4.6–12.0]<br>Any mood disorder<br>11.1% vs 38.3% OR 5.0 [3.0–8.2]	
Secnik, 2005, US (36)	4,504 (2,896; 64.3%)	18≤	ICD-9	ICD-9	Case-control study	General sample – HPM database	(2,252/2,252)	Bipolar disorder 0.58% vs 4.48%<br>Depression 2.93% vs 17.10%	
Clinical sample									
Woon and Zakaria, 2019, Malaysia (39)	120 (133; 94.2%)	18-65	CAADID	M.I.N.I	Cross-sectional study	Psychiatric sample	15.8% (101/19)	Manic/hypomanic episode, lifetime<br>8 (7.9%) vs 8 (42.1%)	
Roncero et al, 2019, Spain (35)	726 (Male 72.5%)	18≤	ASRS (14≤)	DSM-IV-TR	Cross-sectional study	Psychiatric sample – treatment seeking AUD patients	21.1% (573/153)	Mood disorder<br>24.5% vs 49%<br>χ^2 = 32.87, OR 2.95 [2.2, 4.3]	
Leung and Chan, 2017, Hong Kong (31)	254 (28.7%)	18-64	ASRS-v1.1 Symptom Checklist≥17 & SDS ≥5 (Screening) + DIVA 2.0 (Diagnosis)	DSM-5	Cross-sectional cohort study	Psychiatric sample – clinical outpatients	19.3% (49/205)	ADHD-combined type vs Non-ADHD<br>Bipolar disorder 2.0% vs 15.0%<br>OR = 8.87 (1.83-42.9)	
Gorlin et al,	1,134	Mean age	DSM-IV	SCID	Cross-sectional	Psychiatric	18.0%	Major depressive disorder	

2016, US (28)	(476; 42%)	39.7 (SD 14.4)	based semi-structured clinical interview		study	sample - clinical outpatients	(204/903)	39.6% vs 29.4% OR=0. 69 (.49–.96)<br>Bipolar disorder<br>3.4% vs 7.4% OR=2.14 (1.09–4.02)	
Fatséas et al, 2016, France (27)	217 (66.4%)	Mean age 37.7<br>(SD 10.6)	CAADID	DSM-IV for SUD<br>SCIDII for BPD<br>M.I.N.I. for others	Cross-sectional cohort study	Psychiatric sample – addiction clinical outpatients	23.0% (50/167)	Current mood disorders<br>36.8% vs 54.0% 0.030	
van Emmerik-van Oortmerssen et al, 2014, Australia, Belgium, France, Hungary, Netherlands, Norway, Spain, Sweden, Switzerland, US (IASP study) (38)	1,205 (ADHD – 73.1% male, ADHD + 75.6 male)	18–65	CAADID	MINI Plus<br>SCID-II	Cross-sectional study	Psychiatric sample - treatment-seeking SUD patients	13.9% (168/1,037)	Current Depression - alcohol<br>15.3% vs 39.7% OR 4.1 [2.1–7.8]<br>Current (hypo)mania<br>4.1% vs 14.9% OR 4.3 [2.1–8.7]	ISAP-International ADHD in Substance use disorder Prevalence
Duran et al, 2014, Turkey (26)	246	Mean age ADHD+ 27.38 ± 8.3<br>ADHD – 30.97 ± 8.6	WURS score >36 & Turgay's Adult ADD/ADHD	SCID-I-CV, SCID-II	Cross-sectional study	Psychiatric sample - clinical outpatients	15.9% (39/207)	Dysthymic Disorder<br>12 (5.8%) vs 6 (15.4%) $\chi 2$ = 25.81	

			Evaluation Scale						
Perugi et al, 2013, Italy (34)	96 (57; 59.4%)	Mean age ADHD+ 38.5 (SD 13.5) ADHD- 43.2 (SD 13.4)	ASRS v 1.1 (+), & prior age 7 with ADHD sx	DSM-IV	Cross-sectional observation study	Psychiatric sample - Bipolar I, II disorder diagnosed	19.8% (19/77)	BD I mixed state<br>14 (18.2%) vs 10 (52.6%) χ2= 9.6<br>BD I mania 13<br>(16.9%) vs 0 (0%) χ2= 3.7<br>BD I remission 15 (19.5%) vs 0 (0%) χ2= 0.1	
Ceraudo et al, 2012, Italy (25)	119 (81; 68.1%)	Mean age ADHD+ 35.10 (SD 7.66) ADHD- 34.74 (SD 8.46)	ASRS-S v 1.1 (+)	DCTC (Diagnostic, Clinical and Therapeutic Checklist)	Cross-sectional study	Psychiatric sample – SUD outpatients	18.35% (20/89)	Bipolar Disorder<br>38 (43.2%) vs 16 (80.0%) χ2= 8.84<br>Mixed/Manic<br>15 (16.9%) vs 8 (40.0%) χ2= 3.29	

OR : Odd Ratio, PR : Prevalence Ratio, NA : Not available (not identified in article)
SUD : Substance Use Disorder, AUD: Alcohol use disorder, BPD : Borderline Personality Disorder BD I : Bipolar I disorder
WHO-CIDI : World Health Organization version of the Composite International Diagnostic Interview, CCHS-MH : Community Health Survey–Mental Health, M.I.N.I : Mini-International Neuropsychiatric Interview, ASRS-S: Adult Self-Report Scale-Screener, ASRS: Adult Self-Report Scale, CIDI: Composite International Diagnostic Interview, K-SADS : Kiddie Schedule for Affective Disorders and Schizophrenia, SCID : Structured Clinical Interview for DSM-IV, SCID-I : Structured Clinical Interview for DSM-IV Axis I Disorders, SCID-I-CV : Structured Clinical Interview for DSM-IV Axis I Disorders, Clinician Version, SCID-II : Structured Clinical Interview for DSM-IV Axis II Disorders, WURS : Wender Utah Rating Scale, WURS-k : German short form of the Wender Utah rating scale, BADDS : Brown attention deficit disorder scale, DIS-IV : Diagnostic Interview Schedule for DSM-IV, ACDS : Adult ADHD Clinical Diagnostic Scale, ASHD-RS : ADHD Rating Scale, CAADID : Conner's Adult ADHD Diagnostic Interview for DSM-IV

Table 2. Studies comparing the prevalence of anxiety and related disorder between non-ADHD and ADHD subjects									
Author, year, country	N (Male; %)	Age	Assessment of ADHD	Assessment of comorbid psychiatric disorder	Design	Sample	Prev.of ADHD(%) (non-ADHD/ADHD)	Findings comparing non-ADHD and ADHD and prevalence of comorbid psychiatric disorders<br>non-ADHD, n (%) vs ADHD, n (%)	Comment
General sample									
Solberg et al, 2018, Norway (37)	1,701,206 (871,330; 51.2%)	18≤	ADHD medication at adult or ADHD diagnosis registered	ICD-10	Cross-sectional study	General sample	2.4% (40,103/1,661,103)	Anxiety Disorders<br>Women 54,479 (6.7%) vs 4,676 (26.3%)<br>Men 28,364 (3.3%) vs 4,054 (18.2%)	
Chen et al, 2018, Norway (25)	5,551,807 (2,820,859; 50.81%)	18-64	ICD-9: 314; ICD-10: F90 diagnosis	ICD	cross-sectional study	General sample	1.1% (61,129/5,490,678)	Anxiety<br>PR= 9.12 (9.04–9.21)	
Hesson and Fowler, 2018,	16,957	20-64	Self-report of ADHD	WHO-CIDI modified for	Case-control study	General sample	2.9% (NA)	Generalized anxiety disorder 15 (3.1%) vs 73 (15.1%) χ2 =	

Canada (29)			(diagnosed by a health professional)	the needs of CCHS-MH		-national mental health survey		42.30	
Yoshimasu et al, 2016, US (16)	5,718	Mean age ADHD 30.2 (SD 1.9) Non-ADHD controls 30.2 (SD 2.0)	childhood-identified ADHD with M.I.N.I (+)	M.I.N.I.	Case-control study	General population – Birth cohort sample	NA, (68/335)	PTSD 3 (0.9%) vs 6 (8.8%) OR adj. 10.0 [2.9, 35.0] Social phobia-current 4 (1.2%) vs 10 (14.7%) OR adj 12.8 [4.2, 39.4] OCD 8 (2.4%) vs 14 (20.6%) OR adj 8.0 [3.3, 19.2] Generalized anxiety disorder 30 (9.0%) vs 22 (32.4%) OR adj 4.7 [2.4, 9.0] Panic disorder – lifetime 17 (5.1) vs 9 (13.2) OR adj 2.6 [1.1, 6.2]	
Park et al, 2011, South Korea (33)	6,081 ADHD+ 59.4% ADHD- 50.5%	18-59	ASRS-S v 1.1 (+)	K-CIDI (Korean Ver. of CIDI)	Epidemiological study	General sample	1.1% (69/6,012)	Any anxiety disorder 6.3% vs 25.7% OR 5.46 [3.11–9.57] OCD 0.6% vs 4.3% OR 8.26 [2.51–27.26] PTSD 1.2% vs 7.2% OR 8.13[3.26–20.32] Social phobia 0.5% vs 11.4% OR 7.57[1.92–29.83]	

								Specific phobia<br>3.9% vs 11.4%<br>OR 3.31 [1.52–7.18]<br>Somatoform disorder<br>1.1% vs 4.3%<br>OR 4.30 [1.22–15.12]	
Miller et al, 2007, US (32)	363<br>(185, 51.0%)	18-37	K–SADS & structured interview	SCID–I, SCID-II	Case-control study	General sample-Recruited ADHD vs control group	NA, (152/211)	Anxiety disorder χ2= 8.81	
Kessler et al, 2006, US (30)	3,199	18-44	DIS-IV for childhood pathology<br>& ACDS v 1.2 (ADHD-RS)	CIDI	Epidemiologic study	General sample – national survey	2.6%<br>(NA)	GAD 2.6% vs 8.0%<br>OR 3.2 [1.5–6.9]<br>PTSD 3.3% vs 11.9%<br>OR 3.9 [2.1–7.3]<br>Panic disorder 3.1% vs 8.9% OR 3.0 [1.6–75.9]<br>Agoraphobia 0.7% vs 4.0% OR 5.5 [1.6-18.5]<br>Specific phobia 9.5% vs 22.7% OR 2.8 [1.7-4.6]<br>Social Phobia<br>7.8% vs 29.3% OR 4.9 [3.1-7.6]<br>Any anxiety disorder 19.5% vs 47.1% OR 3.7 [2.4–5.5]	

Secnik, 2005, US (36)	4,504 (2,896; 64.3%)	18≤	ICD-9	ICD-9	Case-control study	General sample – HPM database	NA (2,252/2,252)	Anxiety disorder 3.46% vs 13.77%	HPM : Health and Productivity Management
Clinical sample									
El Ayoubi et al, 2020, France (43)	551 (462, 83.8%)	18≤	Both ASRS-S v1.1(+) and WURS (26≤)	PCL-5 for PTSD	Cross-sectional study	Psychiatric inpatients with AUD	19.8% (442/109)	PTSD<br>179 (40%) vs 91 (84%) χ^2 = 64.7	
Woon and Zakaria, 2019, Malaysia (39)	120 (133, 94.2%)	18-65	CAADID	M.I.N.I	Cross-sectional study	Psychiatric sample - Forensic ward inpatient	15.8% (101/19)	Generalized anxiety disorder<br>20 (19.8%) vs 9 (47.4%)	
Roncero et al, 2019, Spain (35)	726 (Male 72.5%)	18≤	ASRS (14≤)	DSM-IV-TR	Cross-sectional study	Psychiatric patients – treatment seeking AUD patients	21.1% (573/153)	Anxiety disorder<br>10.5% vs 25.8% χ^2 = 23.5<br>OR 2.95 [1.88, 4.64]	
Reyes et al, 2019, US (42)	472 (305, 64.6%)	18-80	PRISM	PRISM	Cross-sectional study	Psychiatric sample – inpatient & outpatient	6.36% (30/442)	Anxiety disorders, current<br>95 (21.5%) vs 14 (46.7%)	

						with DSM-IV-TR AUD diagnosis			
Gorlin et al, 2016, US (28)	1,134 (476, 42%)	Mean age 39.7 (SD 14.4)	DSM-IV based semi-structured clinical interview	SCID	Cross-sectional study	Psychiatric sample-clinical outpatient	18.0% (204/903)	Social phobia 28.7% vs 38.2% OR=1.46 (1.05–2.01)<br>Any adjustment disorder 9.4% vs 3.9%<br>OR = 0.41 (0.18–0.82)	
Retz et al, 2016, Germany (12)	163 (141,86.5%)	Mean age 40.2 (SD 9.4)	DSM-5 & WURS-k ≥ 30	ICD-10	Cross-sectional study	Psychiatric sample – GD dx according to ICD-10	25.2% (41-current ADHD/122)	Stress and adjustment disorders<br>14 (8.6%) vs 7 (17.1%) $\chi2$ = 5.70	
Karaahmet et al, 2013, Turkey (40)	90 (48, 53.3%)	18 ≤	Turgay's Adult ADD/ADHD Evaluation Inventory & WURS	SCID-I	Cross-sectional study	Psychiatric sample-Bipolar disorder diagnosed	23.3% (21/69)	OCD<br>6 (10.7%) vs 4 (19.0%)<br>Panic disorder<br>3 (5.4%) vs 5 (23.8%)	
Incarcerated sample									
Moore et al, 2016, Australia (41)	88 (76%)	18-72	ASRS-S (+) & M.I.N.I plus (+)	M.I.N.I plus, PDQ-4, SCID-II	Cross-sectional study	Incarcerated sample	17.0% (15/73)	Social phobia 15.1% vs 46.7% OR=4.39 [1.10, 17.56]	

OR : Odd Ratio, NA : Not available (not identified in article)

PTSD : Post-traumatic stress disorder, OCD : Obsessive-compulsive disorder, GAD : Generalized anxiety disorder, AUD : Alcohol used disorder, GD : Gambling disorder

WHO-CIDI : World Health Organization version of the Composite International Diagnostic Interview, CCHS-MH : Community Health Survey–Mental Health, M.I.N.I : Mini-International Neuropsychiatric Interview, ASRS-S: Adult Self-Report Scale-Screener, ASRS: Adult Self-Report Scale, CIDI: Composite International Diagnostic Interview, K-SADS : Kiddie Schedule for Affective Disorders and Schizophrenia, SCID-I : Structured Clinical Interview for DSM-IV Axis I Disorders, SCID-II : Structured Clinical Interview for DSM-IV Axis II Disorders, WURS : Wender Utah Rating Scale, WURS-k : German short form of the Wender Utah rating scale, PRISM : Psychiatric research interview for substance and mental disorders, PDQ-4 : Personality disorder diagnostic questionnaire for the DSM-IV

Table 3. Studies comparing the prevalence of substance use disorder and gambling disorder between non-ADHD and ADHD subjects									
Author, year, country	N (Male; %)	Age	Assessment of ADHD	Assessment of comorbid psychiatric disorder	Design	Sample	Prev.of ADHD(%) (non-ADHD/ADHD)	Findings comparing non-ADHD and ADHD and prevalence of comorbid psychiatric disorders non-ADHD, n (%) vs ADHD, n (%)	Comment
General sample									
Cipollone et al, 2020, US (45)	18,913 (16,696; 88.3%)	Mean age 28.72 in non-ADHD, 28.56 in ADHD	ASRS-S (+)	CIDI & CIDI-SAM	Cross -sectional study (All army study)	General sample – Military sample	6.6% (17,674/1,239)	Previous 30-days SUD diagnosis<br>714 (4.04%) vs 211 (17.03%)<br>χ^2 = 515.36<br>Lifetime SUD diagnosis<br>2,639 (14.93%) vs 503 (40.60%)<br>χ^2 =780.16<br>Alcohol use (type 2 - Five or more drinks per day- heavy drinking)<br>2,064 (12.04%) vs 305 (25.10%)<br>χ^2 =172.07	
Capusan et al, 2019, Sweden (44)	18,167 (7,281; 40.08%)	20-45	DSM-IV criteria	SCID-I	Population-based epidemiological study	General population-Swedish Twin Registry	8.8% (1,598/16,569)	Alcohol abuse<br>OR = 1.88 [1.44, 2.46]<br>Alcohol dependence<br>OR = 3.58 [2.86, 4.49]<br>Stimulants<br>OR = 2.45 [1.79, 3.35]<br>Opiates	

								OR = 1.97 [1.65, 2.36]<br>Cannabis<br>OR = 2.19 [1.80, 2.68]<br>Illicit drug use<br>OR =2.27 [1.86, 2.76]<br>Poly-substance use<br>OR = 2.54[2.00, 3.23]<br>Poly-substance use including alcohol<br>OR = 2.78 [2.21, 3.50]	
Chen et al, 2018, Norway (25)	5,551,807 (2,820,859; 50.81%)	18-64	ICD-9: 314; ICD-10: F90 diagnosis	ICD	cross-sectional study	General sample	1.1% (61,129/5,490,678)	SUD<br>PR= 9.74 (9.62–9.86)	
Hesson and Fowler, 2018, Canada (29)	16,957	20-64	Self-report of ADHD (diagnosed by a health professional)	WHO-CIDI modified for the needs of CCHS-MH	Case-control study	General sample -national mental health survey	2.9% (NA)	12-month<br>Alcohol dependence<br>8 (1.7%) vs 27 (5.6%) χ2 = 10.83 .001<br>Cannabis abuse<br>3 (0.6%) vs 13 (2.7%) χ2 = 6.376 .012<br>Cannabis dependence<br>3 (0.6%) vs 11 (2.3%) χ2 = 4.605 .032<br>Other drug dependence<br>3 (0.6%) vs 17 (3.5%) χ2 = 10.01 .002	
Yoshimasu et al, 2016, US (16)	5,718	Mean age ADHD 30.2 (SD 1.9)	childhood-identified ADHD with	M.I.N.I.	Case-control study	General population –	NA, (68/335)	Alcohol dependence/abuse 51 (15.2%) vs 28 (41.2%) OR adj 3.6 [2.0, 6.7]	

		Non-ADHD controls 30.2 (SD 2.0)	M.I.N.I (+)			Birth cohort sample		Substance dependence/abuse 22 (6.6%) vs 18 (26.5%) OR adj 4.4 [2.1, 9.1]	
Park et al, 2011, South Korea (33)	6,081 ADHD+ 59.4% ADHD- 50.5%	18-59	ASRS-S v 1.1 (+)	K-CIDI (Korean Ver. Of CIDI)	Epidemiological study	General sample	1.1% (69/6,012)	Alcohol abuse/dependence 16.6% vs 30.4% OR 1.97 [1.14–3.38] Nicotine dependence 7.7% vs 20.3% OR 2.81 [1.50–5.29] Pathological gambling 0.7% vs 1.4% OR 8.43 [2.63–26.96]	
Miller et al, 2007, US (32)	363 (185, 51.0%)	18-37	K–SADS & structured interview	SCID–I, SCID-II	Case-control study	General sample-Recruited ADHD vs control group	NA, (152/211)	Any ADHD SUD χ2= 9.22	
Sobanski et al, 2007, Germany (6)	140 (76, 54.3%)	Mean age ADHD+ 36.8 (SD 9.0) ADHD- 39.8 (SD 10.0)	WURS-K & BADDS	SCID-I	Case-control study	General sample -referred ADHD vs control group	NA, (70/70)	Substance related disorders total 5 (7.1%) vs 21 (30.0%) χ2= 12.397 Substances total 2 (2.9%) vs 20 (28.5%) χ2= 17.806 Substance abuse 2 (2.9%) vs 12 (17.1%) χ2= 8.104 Substance dependence 0 (0%) vs 8 (11.4%) χ2= 8.612	

Kessler et al, 2006, US (30)	3,199	18-44	DIS-IV for childhood pathology & ACDS v 1.2 (ADHD-RS)	CIDI	Epidemiologic study	General sample – national survey	2.6% (NA)	Drug dependence 0.1% vs 4.4% OR 7.9 [2.3–27.3] Any substance disorder 5.6 % vs 15.2% OR 3.0 [1.4–6.5]	
Secnik, 2005, US (36)	4,504 (2,896; 64.3%)	18≤	ICD-9	ICD-9	Case-control study	General sample – HPM database	NA, (2,252/2,252)	Drug or alcohol abuse 1.87% vs 5.11%	HPM : Health and Productivity Management
Clinical sample									
Valsecchi et al, 2021, Italy (46)	590 (299, 47.2%)	18-70	ASRS-S v1.1 (+) and DIVA 2.0 both(+)	M.I.N.I Plus	cross-sectional observational study	Psychiatric outpatients	5.12% (590/44)	Substance abuse, lifetime 15.1% vs 29.6% χ2 = 6.34 Substance abuse, actual Present 6.6% vs 25.0% χ2 = 19.06 Substance use, lifetime 30.5% vs 54.6% χ2 = 10.84 .001 Substance use, actual 8.3% vs 29.6% χ2 = 20.93 .000	
Woon and	120	18-65	CAADID	M.I.N.I	Cross-sectional	Psychiatric	15.8%	Alcohol abuse 2 (2.0%) vs 3	

Zakaria, 2019, Malaysia (39)	(133, 94.2%)				study	sample - Forensic ward inpatient	(101/19)	(15.8%) 0.028	
Roncero et al, 2019, Spain (35)	726 (Male 72.5%)	18≤	ASRS (14≤)	DSM-IV-TR	Cross-sectional study	Psychiatric patients – treatment seeking AUD patients	21.1% (573/153)	Cannabis dependence 18% vs 30.9% χ^2 = 12.3 OR 2.04 [1.36, 3.06]<br>Cocaine dependence 24.6% vs 53.3% χ^2 = 46.5 OR 3.5 [2.41, 5.07]<br>Smoking dependence 72.2% vs 82.9% χ^2 = 6.9 OR 1.86 [1.16, 2.98]	
Reyes et al, 2019, US (42)	472 (305, 64.6%)	18-80	PRISM	PRISM	Cross-sectional study	Psychiatric sample – inpatient & outpatient with DSM-IV-TR AUD diagnosis	6.36% (30/442)	Cannabis abuse, Current 41 (9.3%) vs 8 (26.7%)<br>Amphetamine abuse, current 17 (3.9%) vs 4 (13.3%)<br>Opioid abuse, current 9 (2.0%) vs 3 (10.0%)	
Leung and Chan, 2017, Hong Kong (31)	254 (28.7%)	18-64	ASRS-v1.1 ≥17 & SDS ≥5(Screening) + DIVA 2.0 (Diagnosis)	DSM-5	cross-sectional cohort study	Psychiatric sample – clinical outpatients	19.3% (49/205)	Chronic alcohol use (2.4% vs 8.2%)<br>Problematic gambling (1% vs 2%)<br>Active substance use (3.9% vs. 8.2%)	
Retz et al, 2016,	163 (141,86.5%)	Mean age 40.2 (SD 9.4)	DSM-5 & WURS-k ≥	ICD-10	Cross-sectional study	Psychiatric sample –	25.2% (41-current	Substance use disorders 4'50 (30.7%) vs 19 (46.3%) χ2	

Germany (12)			30			GD dx according to ICD-10	ADHD/122)	= 6.50	
Fatséas et al, 2016, France (27)	217 (66.4%)	Mean age 37.7 (SD 10.6)	CAADID	DSM-IV for SUD SCID-II for BPD M.I.N.I for others	Cross-sectional cohort study	Psychiatric sample – addiction outpatient clinic	23.0% (50/167)	Cannabis dependence 25.9% vs 58.0%	
Duran et al, 2014, Turkey (26)	246	Mean age ADHD+ 27.38 ± 8.3 ADHD – 30.97 ± 8.6	WURS score >36 & Turgay's Adult ADD/ADHD Evaluation Scale	SCID-I-CV, SCID-II	Cross-sectional study	Psychiatric sample - outpatient visit patient	15.9% (39/207)	Other Substance Abuse 12 (5.8%) vs 7 (18.0%) $\chi 2$ = 28.81	
Perugi et al, 2013, Italy (34)	96 (57, 59.4%)	Mean age ADHD+ 38.5 (SD 13.5) ADHD- 43.2 (SD 13.4)	ASRS v 1.1 (+), & prior age 7 with ADHD sx	DSM-IV	Cross-sectional observation study	Psychiatric sample- Bipolar I, II disorder diagnosed	19.8% (19/77)	Alcohol 7 (9.1%) vs 5 (26.3%) $\chi 2$= 4.1<br>Substance use disorder 14 (18.2%) vs 8 (42.1%) $\chi 2$= 7.1	
Incarcerated sample									
Moore et al, 2016, Australia (41)	88 (76%)	18-72	ASRS-S (+) & M.I.N.I plus (+)	M.I.N.I plus, PDQ-4, SCID-II	Cross-sectional study	Incarcerated sample	17.0% (15/73)	Benzodiazepine dependence (lifetime) 13.7% vs 53.3 OR = 5.30 ([1.30, 21.72])	
Konstenius et al, 2015, Sweden (23)	96 (All Female)	Mean age 39.7	ASRS-S(+) & CAADID	M.I.N.I	Cross-sectional study	Incarcerated sample- only women	29% (16/40)	Drug dependence 58% vs 100%	

OR : Odd Ratio, PR : Prevalence Ratio, NA : Not available (not identified in article)

SUD : Substance Use Disorder, AUD: Alcohol use disorder, GD : Gambling disorder

CIDI : Composite International Diagnostic Interview, CIDI-SAM : CIDI-Substance Abuse Module, SCID-I : Structured Clinical Interview for DSM-IV Axis I Disorders, WHO-CIDI : World Health Organization version of the Composite International Diagnostic Interview, , M.I.N.I : Mini-International Neuropsychiatric Interview, CAADID : Conner's Adult ADHD Diagnostic Interview for DSM-IV, ASRS-S: Adult Self-Report Scale-Screener, ASRS: Adult Self-Report Scale, BADDS : Brown attention deficit disorder scale, PRISM : Psychiatric research interview for substance and mental disorders SDS: Sheehan Disability Scale, DIVA : Diagnostic Interview for ADHD in Adults, WURS : Wender Utah Rating Scale, WURS-k : German short form of the Wender Utah rating scale, PDQ-4 : Personality disorder diagnostic questionnaire for the DSM-IV, SCID-I-CV : Structured Clinical Interview for DSM-IV Axis I Disorders, Clinician Version

Table 4. Studies comparing the prevalence of personality disorder between non-ADHD and ADHD subjects									
Author, year, country	N (Male; %)	Age	Assessment of ADHD	Assessment of comorbid psychiatric disorder	Design	Sample	Prev.of ADHD(%) (non-ADHD/ADHD)	Findings comparing non-ADHD and ADHD and prevalence of comorbid psychiatric disorders non-ADHD, n (%) vs ADHD, n (%)	Comment
General sample									
Solberg et al, 2018, Norway (37)	1,701,206 (871,330; 51.2%)	18≤	ADHD medication at adult or ADHD diagnosis registere	ICD-10	Cross-sectional study	General sample	2.4% (40,103/1,661,103)	Personality disorder<br>Women 14,079 (1.7%) vs 2,428 (13.6%)<br>Men 8909 (1.1%) vs 2030 (9.1%)	
Yoshimasu et al, 2016, US (16)	5,718	Mean age ADHD 30.2 (SD 1.9) Non-ADHD controls 30.2 (SD 2.0)	M.I.N.I. childhood-identified ADHD with M.I.N.I (+)	M.I.N.I.	Case-control study	General population – Birth cohort sample	NA, (68/335)	Antisocial personality disorder 13 (3.9%) vs 23 (33.8%) OR adj 12.2 [5.3, 27.9]	
Miller et al, 2007, US (32)	363 (185, 51.0%)	18-37	K–SADS & structured interview	SCID–I, SCID-II	Case-control study	General sample-Recruited ADHD vs control group	NA, (152/211)	Any ADHD<br>ASPD<br>$\chi 2= 7.32$	

Secnik, 2005, US (36)	4504 (2,896; 64.3%)	18≤	ICD-9	ICD-9	Case-control study	General sample – HPM database	NA, (2,252/2,252)	Antisocial disorder 0% vs 0.31%<br>Oppositional disorder 0.04% vs 0.53%	HPM – Health and Productivity Management
Clinical sample									
Sánchez-García et al, 2021, Puerto-rico, Hungary, Australia (47)	402 (320, 79.6%)	18-65	CAADID	M.I.N.I Plus	Cross-sectional study	Psychiatric inpatients & outpatients with SUD	35.75% (257/143)	ASPD<br>25.41% vs 53.90% OR 3.26 [2.09, 5.08]<br>BPD<br>20.82% vs 57.45% OR 5.48 [3.40, 8.83]	
Roncero et al, 2019, Spain (35)	726 (Male 72.5%)	18≤	ASRS (14≤)	DSM-IV-TR	Cross-sectional study	Psychiatric patients – treatment seeking AUD patients	21.1% (573/153)	Any personality disorder<br>14.8% vs 37.4 %<br>χ^2 = 38.17 .0001 OR 3.45 [2.29, 5.17]	
Gorlin et al, 2016, US (28)	1,134 (476; 42%)	Mean age 39.7 (SD 14.4)	DSM-IV based semi-structured clinical interview	SCID	Cross-sectional study	Psychiatric sample-clinical outpatient	18.0% (204/903)	Borderline personality disorder<br>7.6% vs 21.9% OR = 3.11 (2.02–4.76)	
Retz et al,	163	Mean age	DSM-5 &	ICD-10	Cross-sectional	Psychiatric	25.2%	Personality disorders	

2016, Germany (12)	(141; 86.5%)	40.2 (SD 9.4)	WURS-k ≥ 30		study	sample – GD dx according to ICD-10	(41-current ADHD/122)	56 (34.4%) vs 27 (65.9%) χ2 = 26.84<br>Cluster B<br>11 (6.7 %) vs 7 (17.1 %) χ2 = 30.49	
Fatséas et al, 2016, France (27)	217 (66.4%)	Mean age 37.7 (SD 10.6)	CAADID	DSM-IV for SUD<br>SCID-II for BPD<br>M.I.N.I for others	Cross-sectional cohort study	Psychiatric sample – addiction outpatient clinic	23.0% (50/167)	Antisocial personality disorder 6.6% vs 26.0%<br>Borderline personality disorder 13.0% vs 34.7%	
van Emmerik-van Oortmerssen et al, 2014, Australia, Belgium, France, Hungary, Netherlands, Norway, Spain, Sweden, Switzerland, US (IASP study) (38)	1,205 (ADHD – 73.1% male, ADHD + 75.6 male)	18–65	CAADID	M.I.N.I Plus<br>SCID-II	Cross-sectional study	Psychiatric sample-treatment-seeking SUD patients	13.9% (168/1,037)	ASPD<br>17.0% vs 51.8% OR 2.8 [1.8–4.2]<br>BPD<br>-alcohol 8.2% vs 34.5% OR 7.0 [3.1–15.6]<br>-drugs 16.7% vs 29.0% OR 3.4 [1.8–6.4]	ISAP-International ADHD in Substance use disorder Prevalence
Incarcerated sample									
Moore et al 2016, Australia (41)	88 (76%)	18-72	ASRS-S (+) & MINI plus (+)	M.I.N.I plus, PDQ-4, SCID-II	Cross-sectional study	Incarcerated sample	17.0% (15/73)	BPD<br>13.7% vs 60.0% OR =7.34 ([1.72, 31.37])	

								ASPD<br>27.4% vs 93.3% OR =26.00 ([2.58, 262.30])	
Konstenius et al, 2015, Sweden (23)	96<br>(All Female)	Mean age 39.7	ASRS-S (+) & CAADID	M.I.N.I	Cross-sectional study	Incarcerated sample-only women	29%<br>(16/40)	ASPD 30% vs 81%	
OR : Odd Ratio, NA : Not available (not identified in article)<br>SUD : Substance Use Disorder, AUD: Alcohol use disorder, GD : Gambling disorder BPD : Borderline Personality Disorder, ASPD : Antisocial personality disorder<br>SCID : Structured Clinical Interview for DSM-IV, M.I.N.I : Mini-International Neuropsychiatric Interview, ASRS-S: Adult Self-Report Scale-Screener, ASRS: Adult Self-Report Scale, CAADID : Conner's Adult ADHD Diagnostic Interview for DSM-IV, WURS-k : German short form of the Wender Utah rating scale, , K-SADS : Kiddie Schedule for Affective Disorders and Schizophrenia, PDQ-4 : Personality disorder diagnostic questionnaire for the DSM-IV									

Discussion

In our systematic review, we included 30 studies conducted over 15 years dealing with the prevalence of psychiatric comorbidities between adults with and without ADHD. To our knowledge, this is the first systematic review comparing the prevalence of comprehensive comorbid psychiatric disorders between adults with and without ADHD and including both general and clinical populations.

In this review, 12 diagnostic tools including clinical diagnostic criteria like DSM or ICD(International Classification of Disease), were used to evaluate adult ADHD. In addition, five diagnostic tools were mainly used for comorbid psychiatric disorders. The most used evaluation tool for adult ADHD was Adult ADHD Self-Report Scale (ASRS) (48, 49) , which was used in 11 studies. Five studies used the ASRS alone to evaluate ADHD in adults, and in the rest of the studies, more than one tool was used together to evaluate ADHD. The next most frequently used evaluation tools were Conner's Adult ADHD Diagnostic Interview for DSM-IV(CAADID) (50) and the Wender Utah Rating Scale (WURS) (51). As a self-report scale, due to its simplicity and cost-effectiveness, ASRS might be preferred by the investigators testing subjects. However, variability in the evaluation tools for adult ADHD was observed and is thought to be due to the lack of established diagnostic criteria for ADHD in adults.

In addition, the prevalence of ADHD in adults varied from 1.1% (33) to 8.8% (44) in general population samples and from 5.12% (46) to 35.75% (47) in psychiatric population samples. A previous systematic review of ADHD prevalence in and adult psychiatric population shows a similar range, from 6.9% to 38.75% (52). In this previous study (52), the authors assumed that

this variety might be due to the diversity of diagnostic methods and the inclusion and exclusion criteria in the studies. Similarly, in our study, the aforementioned variability of diagnostic methodologies for ADHD might have affected this various range of prevalence. In a general population, the estimated mean prevalence rate of ADHD in adults was 2.8% in a previous study (15). Except in two studies included in our review, targeting special populations of army soldiers (45) and twins of Sweden (44), which had higher than estimated prevalence, the range of ADHD prevalence in the general population was 1.1% to 2.9% in our study, similar to that previously observed.

The most frequent comorbid psychiatric disorder in the ADHD group was SUD, ranging between 2.3% and 41.2% in the general population and between 10.0% and 82.9% in the clinical population; two of three studies showed significant prevalence difference between ADHD and non-ADHD subjects. This finding correlates with a previous meta-analysis that reported that almost one out of every four adolescent and adult patients with SUD presents with ADHD (53, 54), which supports SUD as one of the most frequent comorbid psychiatric conditions in adult ADHD. There are some theoretical opinions of shared key characteristics and pathophysiology between ADHD and SUD, like dopaminergic dysregulation of motivational and reward systems, or reduced frontal function including executive functions and response inhibition (55, 56).In addition, considering that childhood ADHD is a prominent risk factor for substance misuse and development of SUD due to the most frequent comorbidities in childhood ADHD, like conduct disorder or oppositional defiant disorder (57), untreated and preserved ADHD in adults might have influenced the cross-sectional difference of prevalence rate between ADHD and non- ADHD patients.

Mood disorders, including depressive disorders and bipolar disorders, were also frequently

observed comorbid psychiatric disorders in ADHD subjects compared to non-ADHD subjects. The estimated prevalence of depressive disorders in the ADHD group ranged from 8.6% to 55% in the general population and 15.4% to 39.7% in the clinical population. Also, the prevalence of bipolar disorder in the ADHD group was estimated at 4.45% to 35.3% in the general population at 7.4% to 80.8% in the clinical population. For depression, previous studies have also shown a higher prevalence of depressive disorders in young adults with ADHD compared to non-ADHD subjects as well as higher risk of suicidal behavior (58, 59). This can be explained by a previous cross-sectional study showing the association between ADHD symptoms and depressive symptoms in young adults as identified by low hedonic responsibility (60).In addition, according to biologic aspects of depression and ADHD, the two disorders might share similar pathophysiologic regions of the brain including decreased activity in the prefrontal (61, 62), amygdala, and hippocampus regions (63-65). Furthermore, 10 studies showed a higher prevalence of bipolar disorder, including current hypomania diagnosed by SCID-II, in ADHD subjects than in non-ADHD subjects. Considering that the worldwide prevalence rate of bipolar disorder is estimated as 1-3% (66, 67), which was similar to that of the non-ADHD general population in our study, the prevalence of bipolar disorder in the ADHD group was greater than 3% in all 10 studies. This finding correlated with previous studies reporting reciprocal high comorbidity rates between ADHD and bipolar disorder, which suggests possible shared genetic effects or diagnostic overlap between the two disorders (68).

In anxiety disorder, almost two of three studies showed a higher prevalence in the ADHD group than the non-ADHD group. The prevalence rate in the ADHD group was estimated to rang e from 4.3% to 47.1% in the general population and from 3.9% to 84% in the clinical population. Only one study of a clinical population dealing with psychiatric outpatients in the US (28),

showed a higher prevalence of adjustment disorder in the non-ADHD group. These findings correlate with previous studies that revealed a high prevalence of anxiety in the adult ADHD population (69, 70). ADHD seems to show different characteristics from anxiety, namely fearlessness and impulsivity. Therefore, various theories have been suggested to explain this phenomenon using developmental or biologic aspects in children and adolescents (71). Similarly, in adults, as far as we know, the two disorders have been related to several common neuroanatomical regions like the dorsolateral prefrontal cortex or the anterior cingulate cortex, which are critically involved with the executive function control network (10). In addition, considering a previous study about increased risk of accidents in ADHD over the lifespan (72), traumatic events might have influenced the higher prevalence of anxiety disorders. From a developmental viewpoint, as in depressive disorders, this frequently higher prevalence of anxiety disorder might represent the social and relational difficulties induced by ADHD.

The estimated prevalence of personality disorders in the ADHD group was ranged from 0.31% to 33.8% in the general population and from 21.9% to 65.95% in the clinical population. Previous studies have reported that personality disorders, mostly cluster B or C personality disorders, are present in almost 50% of adults with ADHD (73). The association between ADHD and personality disorders might be mediated by the symptomatic dimensions of ADHD such as emotional dysregulation and oppositional symptoms (74). In our review, most studies showed a higher prevalence of cluster B personality disorders in ADHD than in non-ADHD groups. Specifically, in the clinical population, more than 20% of adult ADHD subjects were estimated to have comorbid cluster B personality disorder including borderline personality disorder and antisocial personality disorder. Additionally, most clinical population studies included patients diagnosed with substance use disorders, which correlates with previous

observational studies of young male adults with ADHD that revealed associations of antisocial personality disorder with ADHD (75).

Limitations

There are several limiting factors in this review. As mentioned previously, there is significant heterogeneity across studies diagnosing both ADHD and comorbid psychiatric disorders. This prohibited meta-analysis. Furthermore, except for two international studies (38, 47), the included studies were conducted in high-income regions like Europe or North America. For this reason, selection bias may have occurred in our results of the prevalence of comorbid psychiatric disorders in adult ADHD. Considering a previous epidemiologic study investigating cross-national ADHD prevalence in adults (15), the prevalence differed by country income, so our findings might be limited. In addition, we did not differentiate patients according to ADHD or comorbid psychiatric disorder treatment status, which might also have affected the prevalence of comorbid psychiatric disorders. Of the included studies, most explored the prevalence cross-sectionally, so we could not infer a correlation or antecedent relationship between ADHD and comorbidities. Only limited estimates of the associations between ADHD and comorbidities can be provided by our review at the study level.

Conclusion

In conclusion, our findings indicate a higher prevalence of comorbid psychiatric disorders in ADHD subjects compared to non-ADHD subjects, whether they were previously diagnosed with other psychiatric disorders or not. Furthermore, our results suggest a complex association between the multiple comorbidities of ADHD. Given that ADHD is often unrecognized and under-diagnosed in adults, screening for ADHD might be beneficial for patients presenting

multiple psychopathologies including substance abuse, mood disorders, and anxiety disorders. In the future, research on standardization of ADHD diagnosis in adults and its comorbid psychopathologies is required to clarify the aspects to treat and improve the quality of life and the functional and psychosocial impairments of individuals with ADHD. In addition, research on the neurobiological and developmental bases of ADHD and its comorbid psychiatric disorders should continue to improve the understanding of the connectivity and associations between various comorbid psychiatric disorders and ADHD in adults.

Author Contributions

Conceptualization : WMB, YSW, Data Curation & Formal Analysis : WSC, YSW, Writing – Original Draft Preparation : WSC, YSW, Writing – Review & Editing – SMW, HKL, Supervision : WMB

References

1. American Psychiatric Association. Diagnostic and statistical manual of mental disorders: DSM-5. Arlington, VA : American Psychiatric Association (2013)

2. Shaw M, Hodgkins P, Caci H, Young S, Kahle J, Woods AG, et al. A systematic review and analysis of long-term outcomes in attention deficit hyperactivity disorder: effects of treatment and non-treatment. BMC Med. (2012) 10:99. doi: 10.1186/1741-7015-10-99

3. Faraone SV, Biederman J, Mick E. The age-dependent decline of attention deficit hyperactivity disorder: a meta-analysis of follow-up studies. Psychol Med. (2006) 36(2):159-65. doi: 10.1017/s003329170500471x

4. Simon V, Czobor P, Bálint S, Mészáros Á, Bitter I. Prevalence and correlates of adult attention-deficit hyperactivity disorder: meta-analysis. Br J Psychiatry. (2009) 194(3):204-11. doi: 10.1192/bjp.bp.107.048827

5. Polanczyk G, de Lima MS, Horta BL, Biederman J, Rohde LA. The worldwide prevalence of ADHD: a systematic review and metaregression analysis. Am J Psychiatry. (2007) 164(6):942-8. doi: 10.1176/ajp.2007.164.6.942

6. Sobanski E, Brüggemann D, Alm B, Kern S, Deschner M, Schubert T, et al. Psychiatric comorbidity and functional impairment in a clinically referred sample of adults with attention-deficit/hyperactivity disorder (ADHD). Eur Arch Psychiatry Clin Neurosci. (2007) 257(7):371-7. doi: http://dx.doi.org/10.1007/s00406-007-0712-8

7. Faraone SV, Biederman J. Neurobiology of attention-deficit hyperactivity disorder. Biol Psychiatry. (1998) 44(10):951-8. doi: https://doi.org/10.1016/S0006-3223(98)00240-6

8. Seo J-C, Jon D-I, Shim S-H, Sung H-M, Woo YS, Hong J, et al. Prevalence and Comorbidities of Attention Deficit Hyperactivity Disorder Among Adults and Children/Adolescents in Korea. Clin Psychopharmacol NeuroSci. (2022) 20(1):126-34. doi: 10.9758/cpn.2022.20.1.126

9. Biederman J, Faraone SV, Spencer T, Wilens T, Norman D, Lapey KA, et al. Patterns of psychiatric comorbidity, cognition, and psychosocial functioning in adults with attention deficit hyperactivity disorder. Am J Psychiatry. (1993) 150(12):1792-8. doi: 10.1176/ajp.150.12.1792

10. Katzman MA, Bilkey TS, Chokka PR, Fallu A, Klassen LJ. Adult ADHD and comorbid disorders: clinical implications of a dimensional approach. BMC Psychiatry. (2017) 17(1):302. doi: 10.1186/s12888-017-1463-3

11. Fayyad J, de Graaf R, Kessler R, Alonso J, Angermeyer M, Demyttenaere K, et al. Cross-national prevalence and correlates of adult attention-deficit hyperactivity disorder. Br J Psychiatry. (2007) 190:402-9. doi: http://dx.doi.org/10.1192/bjp.bp.106.034389

12. Retz W, Ringling J, Retz-Junginger P, Vogelgesang M, Rösler M. Association of

attention-deficit/hyperactivity disorder with gambling disorder. J Neural Transm. (2016) 123(8):1013-9. doi: http://dx.doi.org/10.1007/s00702-016-1566-x

13. Kooij JJS, Bijlenga D, Salerno L, Jaeschke R, Bitter I, Balázs J, et al. Updated European Consensus Statement on diagnosis and treatment of adult ADHD. Eur Psychiatry. (2019) 56:14-34. doi: 10.1016/j.eurpsy.2018.11.001

14. Raman SR, Man KKC, Bahmanyar S, Berard A, Bilder S, Boukhris T, et al. Trends in attention-deficit hyperactivity disorder medication use: a retrospective observational study using population-based databases. Lancet Psychiatry. (2018) 5(10):824-35. doi: 10.1016/s2215-0366(18)30293-1

15. Fayyad J, Sampson NA, Hwang I, Adamowski T, Aguilar-Gaxiola S, Al-Hamzawi A, et al. The descriptive epidemiology of DSM-IV Adult ADHD in the World Health Organization World Mental Health Surveys. Atten Defic Hyperact Disord. (2017) 9(1):47-65. doi: 10.1007/s12402-016-0208-3

16. Yoshimasu K, Barbaresi WJ, Colligan RC, Voigt RG, Killian JM, Weaver AL, et al. Adults With Persistent ADHD: Gender and Psychiatric Comorbidities—A Population-Based Longitudinal Study. J Atten Disord. (2016) 22(6):535-46. doi: 10.1177/1087054716676342

17. Page MJ, McKenzie JE, Bossuyt PM, Boutron I, Hoffmann TC, Mulrow CD, et al. The PRISMA 2020 statement: an updated guideline for reporting systematic reviews. Syst. Rev. 2021;10(1):89.

18. Andrews G, Slade T, Issakidis C. Deconstructing current comorbidity: data from the Australian National Survey of Mental Health and Well-Being. Br J Psychiatry. (2002) 181:306-14. doi: 10.1192/bjp.181.4.306

19. Bijl RV, Ravelli A, van Zessen G. Prevalence of psychiatric disorder in the general population: results of The Netherlands Mental Health Survey and Incidence Study (NEMESIS). Soc Psychiatry Psychiatr Epidemiol. (1998) 33(12):587-95. doi: 10.1007/s001270050098

20. Plana-Ripoll O, Pedersen CB, Holtz Y, Benros ME, Dalsgaard S, de Jonge P, et al. Exploring Comorbidity Within Mental Disorders Among a Danish National Population. JAMA

Psychiatry. (2019) 76(3):259-70. doi: 10.1001/jamapsychiatry.2018.3658

21. Young S, Moss D, Sedgwick O, Fridman M, Hodgkins P. A meta-analysis of the prevalence of attention deficit hyperactivity disorder in incarcerated populations. Psychol Med. (2015) 45(2):247-58. doi: 10.1017/s0033291714000762

22. Fazel S, Seewald K. Severe mental illness in 33,588 prisoners worldwide: systematic review and meta-regression analysis. Br J Psychiatry. (2012) 200(5):364-73. doi: 10.1192/bjp.bp.111.096370

23. Konstenius M, Larsson H, Lundholm L, Philips B, van de Glind G, Jayaram-Lindström N, et al. An epidemiological study of ADHD, substance use, and comorbid problems in incarcerated women in Sweden. J Atten Disord. (2015) 19(1):44-52. doi: 10.1177/1087054712451126

24. Ceraudo G, Toni C, Vannucchi G, Rizzato S, Casalini F, Dell'Osso L, et al. Is substance use disorder with comorbid adult attention deficit hyperactivity disorder and bipolar disorder a distinct clinical phenotype? Heroin Addict Relat Clin Probl. (2012) 14(3):71-6.

25. Chen Q, Hartman CA, Haavik J, Harro J, Klungsøyr K, Hegvik TA, et al. Common psychiatric and metabolic comorbidity of adult attention-deficit/hyperactivity disorder: A population-based cross-sectional study. PLoS ONE. (2018) 13(9) doi: 10.1371/journal.pone.0204516

26. Duran S, Nurhan F, Ali K, Bilici M, ÇAliskan M. ADHD in Adult Psychiatric Outpatients: Prevalence and Comorbidity: Turkish Journal of Psychiatry. Turk Psikiyatri Dergisi. (2014) 25(2):84-93.

27. Fatséas M, Hurmic H, Serre F, Debrabant R, Daulouède J-P, Denis C, et al. Addiction severity pattern associated with adult and childhood Attention Deficit Hyperactivity Disorder (ADHD) in patients with addictions. Psychiatry Res. (2016) 246:656-62. doi: http://dx.doi.org/10.1016/j.psychres.2016.10.071

28. Gorlin EI, Dalrymple K, Chelminski I, Zimmerman M. Diagnostic profiles of adult psychiatric outpatients with and without attention deficit hyperactivity disorder. Compr

Psychiatry. (2016) 70:90-7. doi: 10.1016/j.comppsych.2016.06.015

29. Hesson J, Fowler K. Prevalence and correlates of self-reported ADD/ADHD in a large national sample of Canadian adults. J Atten Disord. (2018) 22(2):191-200. doi: http://dx.doi.org/10.1177/1087054715573992

30. Kessler RC, Adler L, Barkley R, Biederman J, Conners CK, Demler O, et al. The prevalence and correlates of adult ADHD in the United States: results from the National Comorbidity Survey Replication. Am J Psychiatry. (2006) 163(4):716-23. doi: 10.1176/ajp.2006.163.4.716

31. Leung VMC, Chan LF. A cross-sectional cohort study of prevalence, co-morbidities, and correlates of attention-deficit hyperactivity disorder among adult patients admitted to the Li Ka Shing psychiatric outpatient clinic, Hong Kong. East Asian Arch Psychiatry. (2017) 27(2):63-70.

32. Miller TW, Nigg JT, Faraone SV. Axis I and II comorbidity in adults with ADHD. J Abnorm Psychol. (2007) 116(3):519-28. doi: 10.1037/0021-843x.116.3.519

33. S, Cho MJ, Chang SM, Jeon HJ, Cho S-J, Kim B-S, et al. Prevalence, correlates, and comorbidities of adult ADHD symptoms in Korea: Results of the Korean epidemiologic catchment area study. Psychiatry Res. (2011) 186(2-3):378-83. doi: http://dx.doi.org/10.1016/j.psychres.2010.07.047

34. Perugi G, Ceraudo G, Vannucchi G, Rizzato S, Toni C, Dell'Osso L. Attention Deficit/Hyperactivity Disorder symptoms in Italian bipolar adult patients: A preliminary report. J Affect Disord. (2013) 149(1-3):430-4. doi: 10.1016/j.jad.2012.12.010

35. Roncero C, Ortega L, Pérez-Pazos J, Lligoña A, Abad AC, Gual A, et al. Psychiatric Comorbidity in Treatment-Seeking Alcohol Dependence Patients With and Without ADHD. J attent disord. (2019) 23(12):1497-504. doi: 10.1177/1087054715598841

36. Secnik K, Swensen A, Lage MJ. Comorbidities and Costs of Adult Patients Diagnosed with Attention-Deficit Hyperactivity Disorder. PharmacoEconomics. (2005) 23(1):93-102. doi: http://dx.doi.org/10.2165/00019053-200523010-00008

37. Solberg BS, Halmøy A, Engeland A, Igland J, Haavik J, Klungsøyr K. Gender differences in psychiatric comorbidity: A population- based study of 40 000 adults with attention deficit hyperactivity disorder. Acta Psychiatr Scand. (2018) 137(3):176-86. doi: http://dx.doi.org/10.1111/acps.12845

38. van Emmerik- van Oortmerssen K, van de Glind G, Koeter MWJ, Allsop S, Auriacombe M, Barta C, et al. Psychiatric comorbidity in treatment- seeking substance use disorder patients with and without attention deficit hyperactivity disorder: Results of the IASP study. Addiction. (2014) 109(2):262-72. doi: http://dx.doi.org/10.1111/add.12370

39. Woon LSC, Zakaria H. Adult attention deficit hyperactivity disorder in a Malaysian forensic mental hospital: A cross-sectional study. East Asian Arch Psychiatry. (2019) 29(4):118-23. doi: 10.12809/eaapl851

40. Karaahmet E, Konuk N, Dalkilic A, Saracli O, Atasoy N, Kurçer MA, et al. The comorbidity of adult attention-deficit/hyperactivity disorder in bipolar disorder patients. Compr Psychiatry. (2013) 54(5):549-55. doi: 10.1016/j.comppsych.2012.11.005

41. Moore E, Sunjic S, Kaye S, Archer V, Indig D. Adult ADHD Among NSW Prisoners: Prevalence and Psychiatric Comorbidity. J Attent Disord. (2016) 20(11):958-67. doi: 10.1177/1087054713506263

42. Reyes MM, Schneekloth TD, Hitschfeld MJ, Karpyak VM. Impact of sex and ADHD status on psychiatric comorbidity in treatment-seeking alcoholics. J Attent Disord. (2019) 23(12):1505-13. doi: http://dx.doi.org/10.1177/1087054718807997

43. El Ayoubi H, Brunault P, Barrault S, Maugé D, Baudin G, Ballon N, et al. Posttraumatic Stress Disorder Is Highly Comorbid With Adult ADHD in Alcohol Use Disorder Inpatients. J Attent Disord. (2020):1087054720903363.

44. Capusan AJ, Bendtsen P, Marteinsdottir I, Larsson H. Comorbidity of Adult ADHD and Its Subtypes With Substance Use Disorder in a Large Population-Based Epidemiological Study. J Attent Disord. (2019) 23(12):1416-26. doi: 10.1177/1087054715626511

45. Cipollone G, Gehrman P, Manni C, Pallucchini A, Maremmani AGI, Palagini L, et al.

Exploring the role of caffeine use in adult-ADHD symptom severity of US army soldiers. J Clin Med. (2020) 9(11):1-10. doi: 10.3390/jcm9113788

46. Valsecchi P, Nibbio G, Rosa J, Tamussi E, Turrina C, Sacchetti E, et al. Adult ADHD: Prevalence and Clinical Correlates in a Sample of Italian Psychiatric Outpatients. J Attent Disord. (2021) 25(4):530-9. doi: 10.1177/1087054718819824

47. Sánchez-García NC, González RA, Ramos-Quiroga JA, Van Den Brink W, Luderer M, Blankers M, et al. Attention Deficit Hyperactivity Disorder Increases Nicotine Addiction Severity in Adults Seeking Treatment for Substance Use Disorders: The Role of Personality Disorders. Eur Addict Res. (2020) 26(4-5):191-200. doi: 10.1159/000508545

48. Adler L, Kessler RC, Spencer T. Adult ADHD self-report scale-v1. 1 (ASRS-v1. 1) symptom checklist. New York, NY: World Health Organization. (2003)

49. Kessler RC, Adler L, Ames M, Demler O, Faraone S, Hiripi E, et al. The World Health Organization Adult ADHD Self-Report Scale (ASRS): a short screening scale for use in the general population. Psychol Med. (2005) 35(2):245-56. doi: 10.1017/s0033291704002892

50. Epstein J, Johnson DE, Conners CK. Conners' Adult ADHD Diagnostic Interview for DSM-IV™. (2001)

51. Ward MF, Wender PH, Reimherr FW. The Wender Utah Rating Scale: an aid in the retrospective diagnosis of childhood attention deficit hyperactivity disorder. Am J Psychiatry. (1993) 150(6):885-90. doi: 10.1176/ajp.150.6.885

52. Gerhand S, Saville CW. ADHD prevalence in the psychiatric population. Int J Psychiatry Clin Pract. (2021):1-13.

53. van der Burg D, Crunelle CL, Matthys F, van den Brink W. Diagnosis and treatment of patients with comorbid substance use disorder and adult attention-deficit and hyperactivity disorder: a review of recent publications. Curr Opin Psychiatry. (2019) 32(4)

54. van Emmerik-van Oortmerssen K, van de Glind G, van den Brink W, Smit F, Crunelle CL, Swets M, et al. Prevalence of attention-deficit hyperactivity disorder in substance use

disorder patients: A meta-analysis and meta-regression analysis. Drug Alcohol Depend. (2012) 122(1):11-9. doi: https://doi.org/10.1016/j.drugalcdep.2011.12.007

55. Sharma A, Couture J. A Review of the Pathophysiology, Etiology, and Treatment of Attention-Deficit Hyperactivity Disorder (ADHD). Ann Pharmacother. (2013) 48(2):209-25. doi: 10.1177/1060028013510699

56. Volkow ND, Fowler JS, Wang GJ. Imaging studies on the role of dopamine in cocaine reinforcement and addiction in humans. J Psychopharmacol. (1999) 13(4):337-45. doi: 10.1177/026988119901300406

57. Groenman AP, Janssen TWP, Oosterlaan J. Childhood Psychiatric Disorders as Risk Factor for Subsequent Substance Abuse: A Meta-Analysis. J Am Acad Child Adolesc Psychiatry. (2017) 56(7):556-69. doi: https://doi.org/10.1016/j.jaac.2017.05.004

58. Fitzgerald C, Dalsgaard S, Nordentoft M, Erlangsen A. Suicidal behaviour among persons with attention-deficit hyperactivity disorder. Br J Psychiatry. (2019) 215(4):615-20. doi: 10.1192/bjp.2019.128

59. Babinski DE, Neely KA, Ba DM, Liu G. Depression and suicidal behavior in young adult men and women with ADHD: evidence from claims data. J Clinical Psychiatry. (2020) 81(6):0-.

60. Meinzer MC, Pettit JW, Leventhal AM, Hill RM. Explaining the Covariance Between Attention-Deficit Hyperactivity Disorder Symptoms and Depressive Symptoms: The Role of Hedonic Responsivity. J Clin Psychol. (2012) 68(10):1111-21. doi: https://doi.org/10.1002/jclp.21884

61. Arnsten AFT. The Emerging Neurobiology of Attention Deficit Hyperactivity Disorder: The Key Role of the Prefrontal Association Cortex. J Pediatr. (2009) 154(5):I-S43. doi: 10.1016/j.jpeds.2009.01.018

62. Treadway MT, Waskom ML, Dillon DG, Holmes AJ, Park MTM, Chakravarty MM, et al. Illness progression, recent stress, and morphometry of hippocampal subfields and medial prefrontal cortex in major depression. Biol Psychiatry. (2015) 77(3):285-94. doi:

10.1016/j.biopsych.2014.06.018

63. Perlov E, Philipsen A, Tebartz van Elst L, Ebert D, Henning J, Maier S, et al. Hippocampus and amygdala morphology in adults with attention-deficit hyperactivity disorder. J Psychiatry Neurosci. (2008) 33(6):509-15.

64. Frodl T, Stauber J, Schaaff N, Koutsouleris N, Scheuerecker J, Ewers M, et al. Amygdala reduction in patients with ADHD compared with major depression and healthy volunteers. Acta Psychiatr Scand. (2010) 121(2):111-8. doi: https://doi.org/10.1111/j.1600-0447.2009.01489.x

65. Onnink AMH, Zwiers MP, Hoogman M, Mostert JC, Kan CC, Buitelaar J, et al. Brain alterations in adult ADHD: Effects of gender, treatment and comorbid depression. Eur Neuropsychopharmacol. (2014) 24(3):397-409. doi: https://doi.org/10.1016/j.euroneuro.2013.11.011

66. Merikangas KR, Akiskal HS, Angst J, Greenberg PE, Hirschfeld RMA, Petukhova M, et al. Lifetime and 12-Month Prevalence of Bipolar Spectrum Disorder in the National Comorbidity Survey Replication. Arch Gen Psychiatry. (2007) 64(5):543-52. doi: 10.1001/archpsyc.64.5.543

67. Merikangas KR, Jin R, He J-P, Kessler RC, Lee S, Sampson NA, et al. Prevalence and Correlates of Bipolar Spectrum Disorder in the World Mental Health Survey Initiative. Arch Gen Psychiatry. (2011) 68(3):241-51. doi: 10.1001/archgenpsychiatry.2011.12

68. Schiweck C, Arteaga-Henriquez G, Aichholzer M, Edwin Thanarajah S, Vargas-Cáceres S, Matura S, et al. Comorbidity of ADHD and adult bipolar disorder: A systematic review and meta-analysis. Neurosci Biobehav Rev. (2021) 124:100-23. doi: https://doi.org/10.1016/j.neubiorev.2021.01.017

69. Piñeiro-Dieguez B, Balanzá-Martínez V, García-García P, Soler-López B. Psychiatric Comorbidity at the Time of Diagnosis in Adults With ADHD: The CAT Study. J Atten Disord. (2016) 20(12):1066-75. doi: 10.1177/1087054713518240

70. Reimherr FW, Marchant BK, Strong RE, Hedges DW, Adler L, Spencer TJ, et al.

Emotional Dysregulation in Adult ADHD and Response to Atomoxetine. Biol Psychiatry. (2005) 58(2):125-31. doi: https://doi.org/10.1016/j.biopsych.2005.04.040

71. Schatz DB, Rostain AL. ADHD with comorbid anxiety: a review of the current literature. J Attent disord. (2006) 10(2):141-9.

72. Brunkhorst-Kanaan N, Libutzki B, Reif A, Larsson H, McNeill RV, Kittel-Schneider S. ADHD and accidents over the life span - A systematic review. Neurosci Biobehav Rev. (2021) 125:582-91. doi: 10.1016/j.neubiorev.2021.02.002

73. Olsen JL, Reimherr FW, Marchant BK, Wender PH, Robison RJ. The effect of personality disorder symptoms on response to treatment with methylphenidate transdermal system in adults with attention-deficit/hyperactivity disorder. Prim Care Companion CNS Disord. (2012) 14(5) doi: 10.4088/PCC.12m01344

74. Reimherr FW, Marchant BK, Williams ED, Strong RE, Halls C, Soni P. Personality disorders in ADHD Part 3: Personality disorder, social adjustment, and their relation to dimensions of adult ADHD. Ann Clin Psychiatry (2010) 22(2):103-12.

75. Estévez N, Dey M, Eich-Höchli D, Foster S, Gmel G, Mohler-Kuo M. Adult attention deficit/hyperactivity disorder and its association with substance use and substance use disorders in young men. Epidemiol Psychiatr Sci. (2016) 25(3):255-66. doi: 10.1017/s2045796015000360

Supporting information

S1. Table. PRISMA checklist (DOC)

Fig 1. PRISMA flow diagram. Flow diagram of the manual screening process for eligible literature

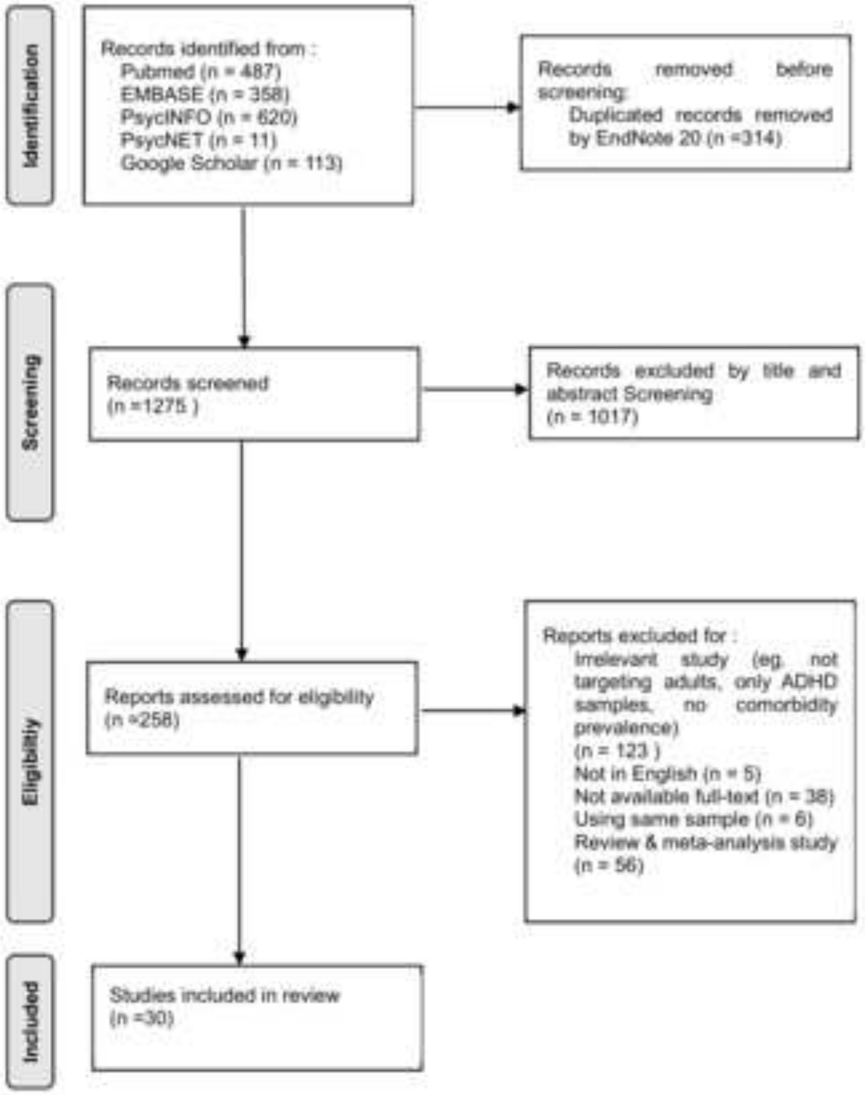

Click here to access/download
Supporting Information
S1_Table.docx
