# Supplementary material for: The prevalence of psychiatric comorbidities in adult ADHD compared with non-ADHD populations: A systematic literature review
Source: PLoS One. 2022 Nov 4;17(11):e0277175. doi: 10.1371/journal.pone.0277175 (PMC9635752; doi:10.1371/journal.pone.0277175)
Supplement: Attachment — Submitted filename: Reviewers comments Revision Plos One.docx [file pone.0277175.s005.docx]

Thank you again for the opportunity to go through the revised submission. The authors have attempted to respond to most queries. I appreciate that. However, they seem to have introduced many grammatical errors in doing so. Also, when I commented on risk of bias assessment, it is important to understand that risk of bias assessment does not only apply to randomized trials, instead there are other tools such as NIH tool for quality check in observation studies or GRADE for overall quality check. I however appreciate the authors effort in using the AMSTAR checklist for quality check.

Finally, I did not find authors’ response to the following comment though it appears that they have tried to keep these in limitations in response to comments from reviewer 1.

Other important points:

Systematic review protocols are required to be registered before you start the review process to avoid duplications and assist in transparency of the work. There are various options for registration such as PROSPERO, the Registry of Systematic Reviews/Meta-Analyses in Research Registry etc.
In the manuscript submitted, I do not see the registration number. Could the authors include the registration number and the registry where the protocol has been registered.
The authors have not described if they have used quality control for the studies included. In other words, risk of bias assessment is one of the most important steps in any systematic review and metanalysis. Also, who did the quality assessment of the studies included?

Overall, the review seems satisfactory to me. All the best.
