# Supplementary material for: The prevalence of psychiatric comorbidities in adult ADHD compared with non-ADHD populations: A systematic literature review
Source: PLoS One. 2022 Nov 4;17(11):e0277175. doi: 10.1371/journal.pone.0277175 (PMC9635752; doi:10.1371/journal.pone.0277175)
Supplement: Attachment — Submitted filename: Response to Reviewers_2nd.docx [file pone.0277175.s006.docx]

**Responses to the Reviewer’s comment**

29 September, 2022

Dear reviewers and editorial staffs of PLOS ONE

We are sincerely grateful for your detailed review and consideration of our manuscript, **“The Prevalence of Psychiatric Comorbidities in Adult ADHD Compared With Non-ADHD Populations : A Systematic Literature Review”**, control number PONE-D-22-17093. Through the comments of first revision process, we could improve the quality of our research. We have additionally revised the manuscript according to the Reviewer’s comments. We performed additional English proofreading, and revised the Reference section in the style required by PLOS ONE (Vancouver style). We acknowledge that the quality of our manuscript was much improved after revising with the scrutinizing efforts of the reviewers and editors.

The changes within the revised manuscript were highlighted in blue. Point-by-point responses to the reviewers’ comments are provided below.

**Reviewer #1**

1. In my previous comment (Reviewer #1, 4.), the authors responded that

"We appreciate for your suggestion. We updated the searched articles after 22 July 2021. We conducted searches up to August 1, 2022 using the same inclusion & exclusion criteria for each database. As a result, 2 more studies were included to our review. We also updated the flow chart, tables and manuscript. Updated numbers and searching strategy of articles are presented at Supplement 2.” It is great to see that the authors updated the search, which made the findings stay highly up to date. However, this information has not been accurately reflected in the revised manuscript: As seen in the current revised Abstract: 'Thirty studies published before July 2021 were identified and classified according to diagnosis of other psychiatric disorder in those with ADHD.' Methods: '...publications regarding the epidemiology and prevalence rate of comorbidities of adult ADHD published from 1 January 1990 to 22 July 2021.'

**Author’s response :** We appreciate the reviewer’s comment. We have confirmed the error in our manuscript. We reflected the updated information and revised the Abstract, Method, Results, and Discussion section of our manuscript. Thank you for the opportunity to correct the mistake according to the reviewer's point. The revised text is shown below :

**Abstract**

…Thirty-two studies published before August 2022 were identified and classified according to diagnosis of other psychiatric disorder in those with ADHD.

**Method**

…We searched electronic libraries of PubMed, EMBASE, PsycINFO, PsycNET, and Google Scholar for publications regarding the epidemiology and prevalence rate of comorbidities of adult ADHD published from 1 January 1990 to 1 August 2022.

**Results**

…Thus, 32 studies of comparing the prevalence rates of comorbid psychiatric disorders between ADHD and non-ADHD adult subjects were selected for systematic review.

…Of the 32 studies comparing the prevalence of comorbid psychiatric disorder between subjects with and without adult ADHD, according to our classification criteria, 11 studies involved general populations, 18 studies included psychiatric populations, and three studies focused on incarcerated populations.

…The most used evaluation tool for adult ADHD was Adult ADHD Self-Report Scale (ASRS) (25, 26) , which was used in 12 studies. Five studies used the ASRS alone to evaluate ADHD in adults, and the rest of the studies used more than one tool to evaluate ADHD.

**Discussion**

…In our systematic review, we included 32 studies conducted over 16 years dealing with the prevalence of psychiatric comorbidities between adults with and without ADHD.

2. In the limitation section, the authors added the new text

'When our review was evaluated by AMSTAR-2 (85), which evaluates the quality of systematic review studies, our review was rated as low quality review.'

I appreciate that the authors provided a detailed response to address my comment (Reviewer #1, 5) regarding the limitation of not evaluating the risk of bias on each study; and provided a detailed explanation within the manuscript, '...Additionally, we did not assess the risk of bias in each studies. In order to include as many studies as possible to reflect broad and various studies of different countries, populations and comorbid psychiatric disorders, we skipped the risk of bias assessment....' The authors further evaluated their review based on AMSTAR-2 and concluded that their current study as 'a low quality review'. According to Shea et al., 2017, a low quality review means "the review has a critical flaw and may not provide an accurate and comprehensive summary of the available studies that address the question of interest". At the moment, without a detailed elaboration on what AMSTAR-2 evaluates, what it actually means 'as a low quality review', what the 'critical flaw' is in the current review, and how it 'may not provide an accurate and comprehensive summary of the available studies that address the question of interest'; it may confuse the readers and devalues the merit of the current review unnecessarily. As the authors have already adequately justified why the risk of bias assessment was not conducted, I suggest the authors to re-consider the necessity and value of including this additional information.

**Author’s response :** We appreciate the reviewer’s thoughtful suggestion. We agree with the reviewer’s opinion that current revised manuscript can confuse the readers. After discussion with other authors, we have decided to delete the sentence which could devalue the meaning of current study. This will clarify the meaning of our study. Thank you for the reviewer’s comment.

3. A thorough proofread and editing is required for the updated text to enhance readability: eg.

i) In the method section, data collection,

'Microsoft Excel was used to develop a data extraction sheet, and the whole included full-text

articles were reviewed by two researchers (WSC, YSW) in duplicate who also

conducted the initial data search and study selection process.'

While the authors explained the process clearly in their response (Reviewer #1, 2.):

'...We wanted to mean that both two researchers reviewed all article which was selected by them in any order...' However, the expression in the manuscript still seems to be unclear, especially after putting the words 'in duplicate'. Suggest to reword.

Ii) Study selection section, 'a full-text review was performed to define eligibility of each studies.'; discussion section, ''...Additionally, we did not assess the risk of bias in each studies....'

Should it be each study?

Iii) In discussion, 'The most frequent comorbid psychiatric disorder in the ADHD group was SUD, it’s prevalence ranging from between 2.3% and 41.2% in the general population and between 10.0% and 82.9% in the clinical population',

Should it be ranging from xx to xx?

iv) In conclusion section updated text, 'Which could help for differential diagnosis and providing earlier treatment to adult ADHD individual.'

It seems to be an dependable clause rather than a complete sentence.

**Author’s response :** We appreciate the reviewer’s comment. We confirmed the part mentioned by the reviewer and revised the sentence in manuscript. Also, we received additional English proofreading for the sentences updated to the manuscript. We have attached a confirmation of English proofreading. Thank you for the reviewer’s comment. The revised text is shown below :

**Study selection**

…The initial inclusion/exclusion review was based on titles and abstracts ; if the relevance of the article was unclear, a full-text review was performed to determine the eligibility of each study.

**Data collection process**

…Microsoft Excel was used to develop a data extraction spreadsheet, and all included full-text articles were reviewed by both researchers (WSC, YSW), who also conducted the initial data search and study selection process.

**Discussion**

…The most frequent comorbid psychiatric disorder in the ADHD group was SUD, it’s prevalence ranging from 2.3% and 41.2% in the general population and between 10.0% and 82.9% in the clinical population;

**Conclusion**

…This standardization will aid in differential diagnosis and allow provision of earlier treatment in adult ADHD.

**Reviewer # 3**

Reviewer #3: Thank you again for the opportunity to go through the revised submission. The authors have attempted to respond to most queries. I appreciate that. However, they seem to have introduced many grammatical errors in doing so. Also, when I commented on risk of bias assessment, it is important to understand that risk of bias assessment does not only apply to randomized trials, instead there are other tools such as NIH tool for quality check in observation studies or GRADE for overall quality check. I however appreciate the authors effort in using the AMSTAR checklist for quality check.

Finally, I did not find authors’ response to the following comment though it appears that they have tried to keep these in limitations in response to comments from reviewer 1.

Other important points:

Systematic review protocols are required to be registered before you start the review process to avoid duplications and assist in transparency of the work. There are various options for registration such as PROSPERO, the Registry of Systematic Reviews/Meta-Analyses in Research Registry etc.

In the manuscript submitted, I do not see the registration number. Could the authors include the registration number and the registry where the protocol has been registered.

The authors have not described if they have used quality control for the studies included. In other words, risk of bias assessment is one of the most important steps in any systematic review and meta-analysis. Also, who did the quality assessment of the studies included?

Overall, the review seems satisfactory to me. All the best.

**Author’s response** : We appreciate the reviewer’s thoughtful comments. We performed additional English proofreading for sentences added after first revision. We have attached a confirmation of English proofreading. Also, it was our mistake for not responding to reviewer’s comments about the registration and risk of bias assessment. We apologize for causing the reviewer any inconvenience. The following paragraph is answer for reviewer’s comment :

As answered to Reviewer #1 in the previous Response to Reviewers, we also agree that the registration process and risk of bias assessment are important for systematic review. However, due to our study was systematic literature review scoping for summarizing and providing overview about prevalence of comorbid psychiatric disorders in adult ADHD, we could not register our study in PROSPERO. This point is important limitation of this study, so we presented this points in limitation section. Also, in our review, we did not performed meta-analysis or statistical synthesis with extracted data, but we summarized and re-arranged the prevalence of comorbid psychiatric disorders in multiple studies comparing ADHD and non-ADHD adults to examine the trend of the difference of each comorbid psychiatric disorders. In order to include as many studies as possible to reflect broad and various studies of different countries, populations and comorbid psychiatric disorders, we did not assess the quality or risk of bias of each included study. This is also why we presented our study as a systematic literature review. We presented this points in Limitation section of our manuscript. We hope that this could be the explanation for not assessing risk of bias of each study which could affect the limited results of our review.
