# Supplementary material for: The prevalence of psychiatric comorbidities in adult ADHD compared with non-ADHD populations: A systematic literature review
Source: PLoS One. 2022 Nov 4;17(11):e0277175. doi: 10.1371/journal.pone.0277175 (PMC9635752; doi:10.1371/journal.pone.0277175)
Supplement: Attachment — Submitted filename: Response to Reviewers_3rd.docx [file pone.0277175.s007.docx]

**Responses to the Reviewer’s comment**

20 October, 2022

Dear reviewers and editorial staffs of PLOS ONE

We are sincerely grateful for your thorough consideration and scrutiny of our manuscript, **“The Prevalence of Psychiatric Comorbidities in Adult ADHD Compared With Non-ADHD Populations : A Systematic Literature Review”**, control number PONE-D-22-17093R2. Our study is a systematic literature review of recent studies dealing with adults with ADHD which compares the prevalence of comorbid psychiatric disorders between ADHD and non-ADHD populations. Dealing with the comprehensive prevalence of comorbid psychiatric disorders in adults with ADHD, our study will provide broad scope for understanding of comorbid psychiatric disorders in adults with ADHD to readers.

Through the careful comments made by the editor and reviewers, we were able to improve the quality of our manuscript. We have revised the manuscript according to the editor and reviewer’s suggestions. We conducted an additional proofreading, and corrected some errors in the manuscript including Tables. We hope that our revised manuscript will be considered and accepted for publication in the PLOS ONE in shortly. We acknowledge that the scientific and clinical quality of our manuscript was improved by the scrutinizing efforts of the reviewers and editors.

Point-by-point responses to the editor and reviewers’ comments are provided below.

**For the editor’s additional comments**

In addition, as shown below, I noticed some other sections that need to be corrected.

Study search and data sources section:

"The methodology of the present review followed by Preferred Reporting Items for Systematic Reviews and Meta-Analysis (PRISMA)." should be corrected to "The methodology of the present review followed the Preferred Reporting Items for Systematic Reviews and Meta-Analysis (PRISMA)."

"...based on exclusion criteria of non-relevant articles (e.g., did not focus on adult patients or did not include psychiatric comorbidity data), non-English articles, full text not available, abstract-only papers, articles that were not peer-reviewed." should be "...based on exclusion criteria of non-relevant articles (e.g., did not focus on adult patients or did not include psychiatric comorbidity data), non-English articles, full text not available, abstract-only papers, and articles that were not peer-reviewed."

Results section:

"After the duplicates excluded, an additional 1121 articles were excluded by..." should be "After the duplicates were excluded, an additional 1121 articles were excluded by..."

"Thus, 32 studies of comparing the prevalence rates of comorbid psychiatric disorders between ADHD and non-ADHD adult subjects were selected for systematic review." should be "Thus, 32 studies comparing the prevalence rates of comorbid psychiatric disorders between ADHD and non-ADHD adult subjects were selected for systematic review."

Discussion section:

"This might because the interest in adult ADHD had increased based the results of

longitudinal follow-up studies on children and adolescents with ADHD." should be "This might be because the interest in adult ADHD had increased based on the results of longitudinal follow-up studies on children and adolescents with ADHD."

"The most frequent comorbid psychiatric disorder in the ADHD group was SUD, it's prevalence ranging from..." should be "The most frequent comorbid psychiatric disorder in the ADHD group was SUD, its prevalence ranging from..." or "The most frequent comorbid psychiatric disorder in the ADHD group was SUD, the prevalence of which ranging from..."

"was estimated at 4.45% to 35.3% in the general population at 7.4% to 80.8% in the clinical population." should be "was estimated at 4.45% to 35.3% in the general population and 7.4% to 80.8% in the clinical population."

"...as identified by low hedonic responsibility" should be "...as identified by low hedonic responsivity"

"The prevalence rate in the ADHD group was estimated to rang e from 4.3% to 47.1%" should be "The prevalence rate in the ADHD group was estimated to range from 4.3% to 47.1%"

"Only one study of a clinical population dealing with psychiatric outpatients in the US (34), showed a higher prevalence of adjustment disorder in the non-ADHD group." should be "Only one study of a clinical population dealing with psychiatric outpatients in the US (34) showed a higher prevalence of adjustment disorder in the non-ADHD group."

Table 3:

Capitalize "C" in "childhood-identified ADHD with M.I.N.I (+)"

**Author’s response :** We appreciate the editor’s thoughtful comments. We revised the mentioned text in our manuscript. And also, we fixed some grammatical errors in the table. Thank you for the opportunity to reconfirm the manuscript and the editor’s efforts to improve the quality of our manuscript.

**Reviewer #1**

1. Thanks for the opportunity to re-review the manuscript. The authors have been very responsive to all the comments and concerns. The language expression has been greatly improved. The information of the updated search has been shown accurately throughout the manuscript.

I recommend the current version of the manuscript is at the publication standard in principle, but there are two very minor issues of the revised text that would like to draw the authors' attention.

Under study selection: 'We included all types of except a systematic review or meta-analysis defined by title and method.' ?all types of "research"

Under limitation: 'study.-To include as many studies as possible to reflect broad and various studies of different countries,'. There is a '-' in front of the 'To' in the clean version of copy. I believe that it may be picked up at the final publication stage anyway.

Other than these, it is a very high quality of work!

**Author’s response :** We appreciate the reviewer’s thoughtful comments. We revised the mentioned text in our manuscript. Thank you for the reviewer's compliment and efforts to improve the quality of our manuscript.

**Reviewer # 3**

I have no further comments to the authors. All queries have been reviewed by the authors and I am satisfied with their final revision.

**Author’s response :** We appreciate the reviewer’s thoughtful comments. Thank you for your efforts to improve the quality of our manuscript.
